# Low-/No-Calorie Sweeteners: A Review of Global Intakes

**DOI:** 10.3390/nu10030357

**Published:** 2018-03-15

**Authors:** Danika Martyn, Maryse Darch, Ashley Roberts, Han Youl Lee, Tina Yaqiong Tian, Naoko Kaburagi, Pablo Belmar

**Affiliations:** 1 Intertek Scientific & Regulatory Consultancy, Farnborough, GU14 0 LX, UK; pablo.belmar@intertek.com; 2 Intertek Scientific & Regulatory Consultancy, Mississauga, ON L5N 2X7, Canada; maryse.darch@intertek.com (M.D.); ashley.roberts@intertek.com (A.R.); han.youl.lee@intertek.com (H.Y.L.); 3 Intertek Scientific & Regulatory Consultancy, Beijing 100015, China; tina.yq.tian@Intertek.onmicrosoft.com; 4 Intertek Scientific & Regulatory Consultancy, Tokyo 103-0012, Japan; naoko.kaburagi@intertek.com

**Keywords:** sweeteners, exposure, consumption, presence, high consumers, diabetics, children

## Abstract

The current review examined published data on the intake of all major low-/no-calorie sweeteners—aspartame, acesulfame-K, saccharin, sucralose, cyclamate, thaumatin and steviol glycosides—globally over the last decade. The most detailed and complex exposure assessments were conducted in Europe, following a standardized approach. Japan and Korea similarly had up-to-date and regular intake data available. The data for other Asian countries, Latin America, Australia/New Zealand and global estimates, evaluated by the Joint FAO/WHO Expert Committee on Food Additives (JECFA), while available, were shown to be more limited in terms of design. Overall, the studies conducted since 2008 raised no concerns with respect to exceedance of individual sweetener acceptable daily intake (ADIs) among the general population globally. The data identified do not suggest a shift in exposure over time, with several studies indicating a reduction in intake. However, some data suggest there may have been an increase in the numbers of consumers of low-/no-calorie-sweetened products. Future research should consider a more standardized approach to allow the monitoring of potential changes in exposure based upon events such as sugar reduction recommendations, to ensure there is no shift in intake, particularly for high-risk individuals, including diabetics and children with specific dietary requirements, and to ensure risk management decisions are based on quality intake analyses.

## 1. Introduction

The food and beverage industry over the last several years has been investigating ways to reduce the levels of free sugars within their products to comply with guidelines and regulations, such as those of the World Health Organisation [1], which has made a strong recommendation to reduce the level of sugar in the diet to less than 10%, and preferably as low as 5%. The drive to lower sugar intakes and the potential for sugar substitution with low-/no-calorie sweeteners has subsequently raised questions regarding the trends in the intake of these ingredients and the potential impact in relation to the respective Acceptable Daily Intakes (ADIs). To address these goals, it was considered important to assess up-to-date information on low-/no-calorie sweetener exposure within the global food supply and potential risk of exceeding safety thresholds by consumers.

At its most basic level, the examination of exposure to a substance requires two main inputs–(1) the concentration of the compound of interest in foods; and (2) consumption data for these foods. All exposure assessments require some aspect of modelling, which are based on ‘exposure scenarios’, as defined by the exposure assessors [2]. Authoritative guidelines recommend that a stepwise or tiered approach is used to examine intake, starting from worst-case, crude methods, continuing to more refined approaches only if the preceding assessment indicated a risk in relation to the toxicological level of concern [2,3]. Over recent years, exposure assessment methodologies have progressed from theoretical calculations of potential exposure (such as the Budget Method) to analyses using real-life individual-based food consumption and chemical concentration data. Notably, in the European Union (EU), the European Food Safety Authority (EFSA) recently released an updated version of the Food Additive Intakes Model template (FAIM, Version 2.0) for use as a screening tool by applicants and risk assessors [4], which utilizes individual-level consumption data for EU population groups, versus the previous publicly-available version of this tool (FAIM 1.0, 1.1 [5,6]), which was based on summary statistics.

With each additional input/level of refinement in an exposure assessment calculation, it is possible to consider more realistic patterns of intake for the cohort under examination. This should be clearly defined when developing the exposure scenario. It is essential to ensure that the results obtained are protective of all individuals in a population group. Young children (due to their higher consumption on a body weight basis), brand loyal individuals (due to a higher affinity to brands which may contain greater levels of the compound of interest) and diabetics (due to an increased dietary requirement for sugar replacers) are all groups who are likely at the upper end of exposure to additives, the latter specifically of relevance for low-/no-calorie sweeteners [3,7,8,9,10]. It is therefore essential to examine the robustness and suitability of the assessments used to ensure that they are fit-for-purpose in the context of risk assessment.

As noted above, all exposure scenarios require assumptions to be made by the assessor; as such, uncertainties are inherent. While crude methods can result in considerable overestimates in exposure, the underestimation of intake is a source of concern to risk managers, who must ensure the protection of all individuals. This is a particular consideration for refined assessments, where there is potential for underestimations of intake, for example, brand loyal individuals who may ingest products with a higher than average use level. As such, uncertainties of the exposure model should be assessed and presented alongside the final estimated results [3,11]. This component of the exposure assessment output provides risk managers with key information on the strengths, limitations, and variability of the results, which can guide risk management measures [12,13].

The most recent comprehensive global review of post-market surveillance data on intense sweetener intakes was conducted in 2008 by A.G. Renwick [9]. There is a large volume of additional studies which have been published since this time, which provide valuable information on global intake of newly permitted sweeteners (e.g., steviol glycosides), as well as additives with an established history of use (e.g., aspartame, sucralose). The aim of this paper was, therefore, to conduct a comprehensive review of the globally published exposure estimates for seven low- and no-calorie sweeteners (aspartame, acesulfame-K, saccharin, sucralose, cyclamate, thaumatin and steviol glycosides) since 2008; outlining the trends in intake on a regional basis and the methods utilized therein.

## 2. Materials and Methods

### 2.1. Selection of Intakes Assessments

In order to identify trends in the intake assessment data available globally, a comprehensive literature search was conducted in October 2017 using the electronic search tool, ProQuest Dialog™. A total of 15 databases were searched and included AdisInsight: Trials, AGRICOLA, AGRIS, Allied & Complementary Medicine™, BIOSIS^®^ Toxicology, BIOSIS Previews^®^, CAB ABSTRACTS, Embase^®^, Foodline^®^: SCIENCE, FSTA^®^, Gale Group Health Periodicals Database, Global Health, MEDLINE^®^, NTIS: National Technical Information Service, and ToxFile^®^. The search terms used reflected the substances (aspartame; acesulfame-potassium; cyclamate; saccharin; steviol glycosides/Stevia; sucralose/Splenda; thaumatin, including synonyms, chemical names, trade names, and Chemical Abstract Service (CAS) numbers) and the outcome (acceptable daily intake (ADI); allowable daily intake; estimated daily intake; dietary exposure; consumption estimate; estimate of consumption; tolerable daily intake; intake assessment); and were not limited to the title and abstract. No restrictions with respect to language were imposed for the literature search. Additional searches were conducted using Spanish, Japanese, Korean and Chinese search engines to identify original language publications by government authorities or research groups which were not captured in the primary literature search; in a similar manner to the main literature search, there were no restrictions to the title and abstract. As Renwick [9] summarized the reported intakes of acesulfame-K, aspartame, cyclamate, saccharin, and sucralose, only studies published after 2008 for these sweeteners are presented in the current study.

### 2.2. Presentation of Data

Results are reported as a percentage of the ADI (%ADI) for average (mean or median) and high-level consumers for the population group examined in each study. The main conclusions reported, as well as comments and any uncertainties (assessed qualitatively, if relevant) associated with the models are also reported. The studies are organized according to data available per region (Africa, Asia, Australia/New Zealand, Europe, Latin America, North America, and global), and are listed in ascending order in Table 1, Table 2, Table 3, Table 4 and Table 5. For studies which conducted more than one assessment scenario, the %ADI is presented as a range (minimum reported value-maximum reported value), to incorporate all available intake estimates reported for the population cohort. The %ADI is presented as reported in publications, or was calculated by obtaining the quotient of the reported estimated intake (mg/kg body weight/day) from the respective ADI, and subsequently multiplying by 100%. The ADIs were based on those derived by the Joint Expert Committee on Food Additives (JECFA), except in European studies wherein estimated intakes were compared to ADIs derived by EFSA. Any exceptions to this are noted in the relevant tables. Notably, the ADIs for acesulfame-K and cyclamate derived by EFSA, of 9 and 7 mg/kg body weight/day [14,15], respectively, are more conservative than those derived by JECFA, of 15 and 11 mg/kg body weight/day [16,17], respectively. As a result, the estimated intakes of acesulfame-K and cyclamate can exceed EFSA’s ADI, but not JECFA’s ADI. An ADI for thaumatin has not been specified by EFSA or JECFA, though thaumatin has been accepted for use as a sweetener―available studies have demonstrated that thaumatin is of limited toxicity and is metabolized to innocuous products [18,19].

In addition, the specific intake assessment inputs (i.e., food consumption, chemical concentration data and assessment method) are described for each study in Appendix A. These studies are also organized according to data available per region (Asia, Australia/New Zealand, Europe, Latin America, North America, and global).

## 3. Methodology and Trends in Intake

### 3.1. Africa

No studies conducted in Africa were identified.

### 3.2. Asia

In Asia, a total of 20 studies were identified, two of which were conducted in China [20,21], one among Indian individuals [22], eight conducted in Japan [23,24,25,26,27,28,29,30,31], and nine conducted for Korean population groups [32,33,34,35,36,37,38,39,40]. The main results and conclusions are presented in Table 1. The assessments conducted varied according to country; the findings are therefore discussed separately in the sections that follow.

#### 3.2.1. China

Liu et al. [20] investigated intakes of sodium cyclamate and sodium saccharin from preserved fruit by Chinese female college students who were determined to be high consumers of these foods. The study was conducted using two assessment models. The first utilized a deterministic approach to estimate average intake, considering the mean consumption of these fruits combined with the average concentration of both sweeteners measured. Mean sodium cyclamate and sodium saccharin levels measured in the preserved fruit samples were above their respective maximum permitted levels (MPLs). The intakes did not exceed the ADI but reached almost 95% of this level for sodium cyclamate. The second model used the full range of consumption values combined with the MPL for the sweeteners to determine the 95% confidence interval. The high-level exposure levels ranged from 12.61 to 15.99% of the ADI for sodium cyclamate and 17.33 to 21.99% of the ADI for sodium saccharin, respectively, and, as such, were notably lower than the deterministic approach in the first model and the ADI. The authors noted that the analysis did not take into account potential exposure from the total diet and recognized that sweetener use in preserved fruit should be monitored more closely. The second study [21] utilized consumption data from the China Nutrition and Health Survey (2002), to examine the theoretical exposure to sodium cyclamate by the Chinese population based on the permitted conditions of use for this sweetener (GB 2760-2014 [41]). Average and high level (97.5th percentile) intakes from the different age groups were compared to the ADI derived by EFSA of 7 mg cyclamate/kg body weight/day. There was no exceedance of the ADI at the mean level of intake, however the 97.5th percentile intakes ranged from 78.08%ADI (adults, 18–59 years) to 246.32%ADI (children 2–3 years), indicating that there may be exceedance of the ADI for this sweetener under the conditions of use among high-level consumers. The authors noted that the dietary information was from 2002, and that food consumption patterns may have changed since this time. There was also no examination into the actual use of this sweetener in foods and beverages, which was identified as a source of overestimation by the authors, as the food categories identified in GB 2760-2014 are broad and likely encompass a greater range of foods than those which contain this sweetener [41].

#### 3.2.2. India

In India, an exposure assessment conducted by Singhal and Mathur [22] focused on the intakes of acesulfame-K, aspartame, saccharin and sucralose among a group of assumed high sweetener-consuming individuals, including diabetic and overweight individuals and female college students (*n* = 158). The study examined consumption of sweetener-containing foods as identified in a food label survey conducted in Delhi in 2005/2006, combined with the MPL for the respective sweeteners [42] and industry use levels for table-top sweeteners (permitted for use at *quantum satis*). The results of the assessment suggest no issue with regard to safety for the four sweeteners, with mean intakes determined to be between 0.5 and 22.4% of the respective ADIs; however, there was no investigation into high level intakes. Given the assumed high consuming nature of the individuals investigated, the estimated intakes may be considered as a proxy for high intakes for the general population.

#### 3.2.3. Japan

Most of the studies conducted in the Japanese population were conducted using a market basket approach based on food consumption data from national dietary surveys [24,25,26,27,29,30,31]. The market-based method involves the analysis of compounds in foods as consumed in a typical diet [12]. The chemical concentration data included in Japanese studies were generally based on an average measurement obtained from multiple government- or national-owned research institutes for samples selected based on the consumption of foods from available dietary records [24,25,26,27,29,30,31]. The %ADI reported by the authors was based on the mean concentration of all evaluated samples (including zeros), with the exception of Kawasaki et al. [25] who also assessed exposure considering only sweetener-containing samples. A distinct assessment approach was utilized by Sato et al. [28], who used shipment volume data to estimate intakes using the disappearance data approach for four sweeteners (acesulfame-K, aspartame, saccharin and sucralose). All of the Japanese studies reported very low intakes of the five sweeteners examined (acesulfame K, aspartame, saccharin, sucralose and steviol glycosides), with estimates determined to be less than 1% of the respective ADIs. Based upon the use of a similar methodology in most studies, it was possible to compare exposure estimates in the Japanese population between 2009 and 2016. The estimated intakes of low-/no-calorie sweeteners were generally consistent, with no notable increases or decreases over time (all below 1%ADI). This agrees with the finding of Sato et al. [28] that there has been no significant change in the daily intake by Japanese individuals compared with historical disappearance data (this data was not available for analysis). The studies examined exposure for the total population, considering a default body weight for each population group. There was no estimation of exposure for consumers only, nor did the assessments consider intakes by high-level consumers. However, given the low-level intakes calculated, including those of children, it is unlikely that there would be a concern regarding exposure among high-level consumers.

#### 3.2.4. Korea

The Korean studies examined exposure to six sweeteners (acesulfame-K, aspartame, saccharin, stevia, sucralose and thaumatin). The studies utilized individual-based consumption data from national dietary surveys, namely the Korean National Health and Nutrition Survey (KNHANES) (2005 to 2014), or the Dietary Intake Survey of Infant, Children and Adolescents (one study only [33]). In two studies [33,34,40], intakes were also estimated using theoretical consumption data (i.e., Budget Method and poundage data). Apart from these two screening assessments, the analyses were based on analytical measurements for foods and beverages available in Korean stores and markets. Most studies examined exposure from the total diet, while Lee et al. [33] evaluated intakes from snacks targeted at children, and Kim et al. [37] evaluated intakes from non-alcoholic beverages only. Assessments examined exposure by all age groups as well as average and high-level level (95th percentile) consumers considering both ‘regular’ (non-brand loyal) consumers and consumers determined to be brand loyal to products containing sweeteners (determined by including analytical data for sweetener-containing foods only). The only exceedance identified for the Korean population (excluding the Budget Method calculations) was for sucralose at the 95th percentile of intake when food consumption data was combined with the mean sweetener concentration for positive samples [34]. The exceedance was calculated to be up to 118%ADI among individuals of all ages >1 year. Subsequent assessments, which used the same methodology as Lee et al. [39] and Kim et al. [42] calculated significantly lower intakes of 7.3% and 12.5%, respectively, of the ADI among high-level consumers for this sweetener. Reductions in intake were noted for acesulfame-K, aspartame and saccharin at both mean and high levels of intake in the studies conducted by Lee et al. [39] and Kim et al. [40] when compared with earlier research by Ha et al. [34,35]: at 1.7–5.2%ADI and 6.3–20.1%ADI (2017) versus 4.0–23.3%ADI and 14.0–66.0%ADI (2013) (acesulfame-K); 0.4–3.2%ADI and 1.7–10.0%ADI (2017) versus 4.2–20.0%ADI and 14.1–49.5%ADI (2013) (aspartame); and 2.8–5.4%ADI; and 10.6–18.4%ADI (2017) versus 7.2–24.8%ADI and 16.0–50.4%ADI (2013) (saccharin). The methods used in these studies were comparable (i.e., national food consumption data for all ages, with the inclusion of positive samples only), which may suggest changes in food consumption patterns (recorded using KNHANES 2010–2014 versus KNHANES 2009) or changes in sweetener concentration levels (measured in 2015–2016 versus 2013) since the publications by Ha and colleagues.

A simple distribution model used by Choi et al. [32] to examine exposure to several sweeteners was repeated by Suh and Choi [36] and Suh et al. [38] for acesulfame-K, aspartame, saccharin and sucralose. Minor differences in the methodologies, such as the types of foods examined, may have impacted the results; however, overall, lower intakes were reported in the 2013 and 2014 publications for both the means and high levels, compared with those in the 2011 publication: 0.09% and 5.08%ADI (2014) versus 4.9% and 14.6%ADI (2011) for acesulfame-K; 0.15% and 6.28%ADI (2014) versus 4.9% and 15.8%ADI (2011) for aspartame; 1.18% and 5.29%ADI (2013) versus 7.5% and 20%ADI (2011) for saccharin; and 0.55% and 15.66%ADI (2013) versus 8.6% and 23.7%ADI (2011) for sucralose. As with all comparisons made between individual studies, it is important to interpret findings with caution; however, as a general note, intakes were well below the respective ADIs, and there is a general trend for a reduction in intakes among Korean individuals in more recent assessments.

### 3.3. Australia/New Zealand

Food Standards Australia New Zealand (FSANZ) has recently evaluated the exposure to steviol glycosides and acesulfame-K in response to proposals to increase the MPLs for these food additives in various foods and beverages [43,44,45,46,47,48]. In both assessments, consumption data was based on national dietary surveys conducted in the general Australian and New Zealand populations. Chemical concentration data were based on the existing and proposed MPLs for the two sweeteners. Occurrence data was not considered in either study, although a 30% market uptake was assumed for steviol glycosides (achieved by multiplying the MPLs by 30%) [43,44,45], based on the conclusion by JECFA at its 63rd meeting that the total replacement of dietary sugars by steviol glycosides was likely overestimated exposure by a minimum of 70% [49,50].

For steviol glycosides, the FSANZ [43,44,45] assessment also conducted brand loyal scenarios, wherein 100% of the market uptake (i.e., 100% presence) was assumed for non-alcoholic beverages (flavored drinks and flavored milk products) as these were the top contributors to intake of this sweetener, while 30% of market uptake or presence was assumed for all other categories. At the 90th percentile of intake, the estimated exposure to steviol glycosides in Australian children, aged 2 to 6 years of age, reached, or exceeded, the ADI by up to 10% in the brand loyal scenarios. The ADI was also reached in New Zealand children, aged 5 to 14 years of age, when brand loyalty was assumed for water based flavored drinks. Nonetheless, the FSANZ [43,44,45] concluded that the proposed increase in MPLs for steviol glycosides in frozen desserts and non-alcoholic beverages was not of concern to public health and safety due to the conservative nature of this assessment, namely that the market uptake and brand loyal scenarios likely overestimated intakes.

With respect to the assessment of acesulfame-K intake, assessments were conducted to examine the anticipated intake from chewing gum alone and from cumulative exposure, considering the total diet. FSANZ concluded that there was no safety concern associated with the proposed increase in the MPL in intense sweetened chewing gum as consumer-only intakes among high-level consumers (90th percentile) were considerably lower than the ADI (9 to 15%), even when intakes from the total diet were considered [46,47,48].

### 3.4. Europe

A total of 19 European studies were identified from peer reviewed journals [51,52,53,54,55,56,57,58,59,60,61,62,63,64,65,66,67,68,69]. A further seven studies were identified from authoritative sources [70,71,72,73,74,75,76]. These data examined exposure by various EU population groups to all seven sweeteners of interest. Most of the scientific opinions published by the EFSA examined intake in response to requests for extension of use for specific sweeteners considering the total diet [73,74] or in foods for special medical purposes (FSMPs) [75,76]. Acesulfame-K and aspartame have been the most frequently evaluated sweeteners in Europe, with intakes estimated for both sweeteners in 16 different peer-reviewed publications and in one EFSA scientific opinion.

Most European studies have examined exposure to sweeteners using the ‘Tier 2’ and/or ‘Tier 3’ approach, as defined in European Commission guidelines [7]. Specifically, this involves using individual-based consumption data from national dietary surveys combined with the MPL for the respective sweeteners, in accordance with Regulation 1333/2008 [77] (Tier 2) and/or chemical concentration data (Tier 3). There were some exceptions to this approach, such as the inclusion of summary statistics derived from the EFSA Comprehensive dataset or the FAIM tool [62,68,70,73,74,78] and annual estimated *per capita* beverage consumption data [54,55,59]. Two assessments by the EFSA considered specific consumption scenarios based on the proposed use of sucralose and acesulfame-K in FSMPs by young children [75,76].

Amongst the studies conducted using the Tier 2/Tier 3 approach [58,60,63,64,65,66,67,69], the assessments utilized the full range of consumption values from individuals in the sample population, combined with a single value for the MPL (Tier 2) or actual usage levels (Tier 3), considering a single value (simple distribution) [58,63,69], or the full range of use levels (probabilistic) [60,64,65,66,67].

Some of the more recently published studies also incorporated sweetener ‘occurrence’ or ‘presence’ data for the sweeteners of interest into the model [64,65,66,67,68,69,71,72]. This component of the assessment model involves accounting for the proportion of foods within a given food category which actually contain the sweetener of interest; there were several sources used for this information, namely market research databases, such as Mintel Global New Products database [68], health service records [65,67], food label surveys [64,66,69] or industry reports [52,71,72].

Several of the identified European studies also incorporated market share data, whereby product sales volumes were considered in the exposure assessment, so as to reduce the uncertainty associated with the concentration data incorporated into Tier 3 assessments; however, this was less common [52,71,72].

The majority of the studies identified were conducted for the general population, with intakes calculated for the mean and high-level consumers in all publications. The high-level intake percentile has been most commonly established at the 95th percentile, which aligns with EFSA resources, such as the EFSA Comprehensive Database [79] and the FAIM Tool [4,6]. Several studies examined intakes in specific subgroups, including young children [52,53,56,60,61,62,64,70,71,72,73,74], diabetics [58,63], phenylketonurics (PKU) or subjects with severe cow’s milk protein allergy (CMPA) (who are consumers of FSMPs that are sweetened to increase palatability) [65,67].

Generally, there was no issue with sweetener intake among the evaluated European population groups. While some exceedances were identified in the scenarios conducted, these were typically reduced below the level of toxicological concern following assessment at more refined tiers of assessment for the various sweeteners examined. The exceptions to this were among young children with PKU, whereby high-level intakes of acesulfame-K exceeded the ADI at both Tiers 2 and 3 in two studies (up to 213% and 105.5%ADI at Tier 3) [65,67]. These assessments were modelled for this at-risk cohort using consumption data for healthy children from national dietary surveys as a surrogate, considering moderate and high-level adherence to clinical recommendations. The lack of cohort-specific consumption data was noted by the authors as a significant source of uncertainty in these studies. EFSA [76] also identified one exceedance of the ADI in young children when examining exposure scenarios for FSMP consumers which contained acesulfame-K in regard to the complete replacement of protein in the diet; this was not identified under partial replacement. The extension of use to these products was approved at up to 9 mg acesulfame-K/g protein, providing 10 g protein per day. When examining total dietary exposure to steviol glycosides, EFSA identified exceedance of the ADI by toddlers (1 to 3 years) [73]; however, as the assessment was based on the MPLs for this additive and exceedance was seen in only one country; this was not identified as a risk for this age group. Another group for whom intakes of sweeteners (acesulfame-K, cyclamate, and steviol glycosides) exceeded the ADI using a Tier 3 model were children (4–6 years of age) with type 1 diabetes [63]. Intakes at the 95th percentile were estimated to exceed the ADI by 16%, 38% and 19% for acesulfame-K, cyclamate, and steviol glycosides, respectively. Authors cautioned the low number of participants in this age group (*n* = 9) as a limitation to this finding and concluded that there is a relatively low chance that children with type 1 diabetes, aged 4 to 18 years, exceed the ADI of different low-/no-calorie sweeteners, but noted that diabetes educators and dieticians should pay attention to the use and consumption of these compounds in foods.

With respect to trends in intake over time in the studies identified, due to differences in the assessment methods (including the age groups studied, country investigated, sweetener of interest, and exposure assessment inputs), it was not possible to compare the data for the majority of the studies identified. Of those for which comparisons can be made, the intake of acesulfame-K, aspartame, and saccharin from specific drinks in Portuguese individuals, decreased slightly when examined by Diogo et al. [59] (range of 0–0.7%ADI for acesulfame-K, 0–0.08%ADI for aspartame, and 0–0.9%ADI for saccharin), versus those calculated by Lino et al. [54] and Lino and Pena [55] (range of 0.6–8.0%ADI for acesulfame-K, 0.07–2.9%ADI for aspartame, and 0–1.28%ADI for saccharin). None of these studies examined cumulative exposure from all beverage types combined, nor total dietary exposure for consumers—with standard volumes used to examine consumption. Although the three studies all used a similar approach to examining exposure, there were differences in the age groups and body weights examined; nonetheless, there was a slight reduction in intakes.

In Norway, intakes of acesulfame-K and aspartame from beverages were calculated in 2008 [52] and in 2014 [72] for all ages. Both studies used actual use level data, and market share information; the Vitenskapskomiteen for Mattrygghet Norwegian Scientific Committee for Food Safety (VKM) [72] also considered presence data in several of the scenarios conducted. The mean intakes of acesulfame-K remained similar, at 3.3–18.9%ADI versus 4.0–18.6%ADI, in 2008 and 2014, respectively; while 95th percentile consumers decreased, from 12.2–72.2%ADI to 10.6–59.3%ADI in the later study. In contrast, the mean and high-level intakes of aspartame increased between 2008 and 2014, from a range of 2.5–5.8%ADI to 3.8–9.6%ADI (mean) and 8.0–21.0%ADI to 10.8–28.6%ADI (95th percentile), respectively. These results are based on beverages only, but are all notably lower than the respective ADIs.

Finally, Vin et al. [60] examined the intakes of acesulfame-K and aspartame (in addition to a range of other food additives) by several European population groups (French, Irish, Italian, and UK). The results for aspartame from this study were similar to the mean and high-level intakes calculated by EFSA in 2013 [70], with mean intakes estimated at 0–30.0%ADI [60] and 1.0–40.8%ADI [70], and high-level intakes at 3.0–83.0%ADI [60], and 3.5–92.3%ADI [70]. Aspartame, as well as acesulfame-K intakes, have more recently been investigated by Buffini et al. [69] (Irish adults) and Le Donne et al. [66] (Italian individuals, aged 3 years and older). Buffini et al. [69] examined intakes for these two sweeteners (alongside several others) using more recent dietary intake data (2011 versus 1999 in Vin et al., 2013 [60]). The later research calculated lower intake values for both sweeteners at both the mean and high-levels of intake, with up to 85%ADI determined in 2013 versus up to 59.3%ADI in 2017 for acesulfame-K, and up 40%ADI in 2013 versus up to 21.6%ADI in 2017 for aspartame. Similarly, for Italian individuals, acesulfame-K intakes were reported at up to 69%ADI by Vin et al. [60] versus up to 27%ADI calculated by Le Donne et al. [66], while aspartame intakes were reported at up to 30% in 2013 and up to 10%ADI in 2017. There were differences in the methodology used for the studies published in 2017 (e.g., different dietary data for Irish adults, older cut-off for Italian individuals, different definitions of high percentiles), which all impact the ability to compare the final estimated results; however, as a general finding, there was a reduction in intakes for both average and high-level consumers for these cohorts, with all intakes considerably lower than the ADIs established for the two sweeteners.

### 3.5. Latin America

In Latin America, a total of seven studies were identified which examined exposure to six sweeteners (acesulfame-K, aspartame, saccharin, sucralose, stevia and cyclamate). One study was identified for Argentinian individuals [81], which was prior to the cut-off applied (2008); however, due to the limited number of studies available for this region, it was included in the current review. Four studies were conducted in Chile [82,83,84,85], and two included participants from Chile as well as Panama, Guatemala, and Peru [86,87].

The objective of six of seven publications identified for Latin America was to examine consumption patterns between individuals with different nutritional statuses with the aim of identifying whether there is an association between the consumption of sweeteners and obesity [82,83,84,85,86,87]. These results have not been considered in the current review, which focuses only on the daily estimates of exposure. The samples investigated in each of the studies were generally small (ranging between 190 and 1229), and the cohorts were not nationally representative. Cagnasso et al. [81] noted as part of their conclusion that additional studies should be conducted using a larger sample to allow the results to be extrapolated to the entire population.

A similar assessment methodology was used in all Latin American publications, whereby consumption data was collected using semi-quantitative food frequency questionnaires (FFQ) for foods and/or beverages identified as containing sweeteners, combined with the sweetener concentration listed on the product label. In several Latin American countries, it is a regulatory requirement to include the specific concentration of any additives present in foods and beverages. This was a valuable source of information, which negated the requirement to depend on maximum permissible levels, thereby providing a more realistic estimate of intakes. However, Cagnasso et al. [81] noted that the use of concentrations declared on product labels, rather than analytical data, was a potential source of uncertainty. In terms of the sources of exposure considered in the assessments, most studies measured intake from the total diet [82,83,84,85,86], whereas others focused only on beverages [81,87]. The majority of studies considered exposure from foods and/or beverages containing sweeteners only–the resulting estimates of intake are therefore representative of consumers only (i.e., sweetener intake by the total population, including non-consumers, were not assessed).

There was a high proportion of consumers of low-/no-calorie sweeteners in the studies conducted in Latin America, with greater than 70% of individuals identified as consumers of products containing sweeteners. This is a common finding for consumption recorded via a FFQ, whereby the intake of foods and beverages of interest is reported over a prolonged period of time—generally one week in the available studies [83,84,86,87]. Irrespective of the percentage of consumers, there was no exceedance of the ADI at the mean level of intake for the six sweeteners examined in any of the studies identified in Latin America—up to 42.8%ADI for the various sweeteners. Most of the studies did not estimate high-level intakes, such as the 90th or 95th percentile, however two articles presented exposures for ‘maximum consumers’ (highest estimated intake of all individuals investigated) of acesulfame-K, aspartame, cyclamate saccharin, and sucralose. These studies estimated intakes of between 33.4% and 172.7% of the ADI (ADI exceeded for cyclamate) [81] and 6.0% to 92.6% of the ADI (ADI nearly was exceeded for acesulfame-K) [82]. Cagnasso et al. [81] reported that 1.5% of students aged 3 to 18 years exceeded the ADI for cyclamate; Hamilton et al. [85] reported that 5.8% of adults and 25% (16 of 63) of diabetic children exceeded ADI for saccharin. Although a similar methodological approach was taken for the various studies conducted, it was not possible to discern patterns in intake over time due to differences in the countries, age groups and specific foods studied, as well as the sweeteners examined.

### 3.6. North America

No assessments were identified in North America that presented actual daily exposure estimates to low-/no-calorie sweeteners; however, a number of studies reported the percentage of consumers [88,89,90,91]. These studies estimated the proportion of United States (US) individuals, aged ≥2 years (participants of US National Center for Health Statistics’ National Health and Nutrition Survey) who reported consumption of food and/or beverages described as containing low-/no-calorie sweeteners. Piernas et al. [90] also reported food and beverage purchases of sweetener-containing foods in US households (Nielsen Homescan 2000–2010).

Based on 2009–2010 National Health and Nutrition Examination Survey (NHANES) data, Fakhouri et al. [88] reported that 20% of the total population surveyed were consumers of diet beverages, and Sylvetsky et al. [91] reported that 25.1% and 41.4% of children and adults in the 2009–2012 release reported consuming food and beverages containing non-nutritive sweeteners. In studies evaluating consumption trends, using the most recent U.S. NHANES cycles [88,89,90], it was observed that the percentage of consumers of low-/no-calorie sweeteners had increased considerably since 1999/2000.

Both Sylvetsky et al. [91] and Piernas et al. [90] indicated that the intakes of low-/no-calorie sweeteners could not be assessed, as actual use levels are not required to be disclosed on food labels in the US.

While no studies were available examining post-market surveillance intakes in North America, potential exposures to steviol glycosides by the US population were modelled by JECFA based on dietary sugar replacement (see below for more details).

### 3.7. Global

Global steviol glycoside intake was evaluated by JECFA in 2008, using the Global Environmental Monitoring System (GEMS)/food consumption database, assuming dietary sugars are completely replaced by steviol glycosides based on its relative sweetness intensity compared with sucrose (200) [92]. The committee also evaluated a similar assessment conducted for the US population, in addition to estimates based on disappearance data supplied by Japan and China. Finally, the committee reviewed published high-level intake estimates of this sweetener in healthy and diabetic children as well as in diabetic adults. Based on the GEMS/food consumption database, the estimated exposure to steviol glycosides (assuming complete replacement of dietary sugar) exceeded the ADI by up to 25%. Similarly, *per capita* intakes in the US (again, assuming complete replacement of dietary sugar) exceeded the ADI by 45%. Nonetheless, JECFA concluded that steviol glycoside intake was highly overestimated in these scenarios (by 70 to 80%) given the assumption of complete replacement of dietary sugars, and, as a result, steviol glycoside intake would not realistically exceed the ADI [92]. In Japan, *per capita* intake of steviol glycosides was below the ADI (*per capita* intake in China was not presented). Similarly, high level intake of steviol glycosides in healthy children, diabetic children, and diabetic adults was below the ADI in the published data.

In 2008, JECFA was requested by the Codex Committee on Food Additives to evaluate the impact of different maximum levels of use of cyclamates in the Codex GSFA Food Category 14.1.4 ‘water-based flavored drinks, including “sport”, “energy” or “electrolyte” drinks and particulated drinks’ [93]. As part of this evaluation, the committee reviewed information received from Australia which provided estimates of exposure to cyclamates in beverages at various use levels, as well as published information from Brazil, Germany, Italy, New Zealand, Spain and the United Kingdom. Most of the mean reported dietary intakes for cyclamates were well below the ADI for this sweetener (0.5% to 40.9%). Some exceedances were noted at high percentiles among children and people with diabetes or on weight control diets and in the older studies examined (when maximum use levels for cyclamates were higher than current provisions), with estimates of up to 162.7%ADI identified. The committee concluded that a maximum use level of 350 mg/kg in ‘water-based flavored drinks’ resulted in dietary exposures for high consumers, including children, that were less than the ADI—this is the maximum use level which has been established for this GSFA category [93].

## 4. Discussion

The current review has examined the most recently available published data on exposure assessments for seven of the most commonly used low-/no-calorie sweeteners globally. Other high-intensity sweeteners, such as Luo Han Guo fruit extracts, advantame and neotame have a more limited use, which precluded their inclusion in the review. The information obtained as part of this review identifies a large variation in the methodologies used and the level of detail available for individual low-/no-calorie sweeteners on a regional basis. Studies were identified which applied the full range of established assessment methodologies, from top-line assessments, to complex, detailed estimations. Cruder methods, known as ‘deterministic models’ or ‘point estimates’ which are quick to run and provide a rough estimation of exposure, allowing the identification of potential exceedances of the toxicological level of concern (e.g., Budget Method) and/or permit an examination of patterns of consumption over time (e.g., disappearance data), were generally used to guide more resource-intensive models. More refined assessments, so-called ‘distributional models’, which incorporate the full distribution of food consumption and/or chemical concentration data and more accurately reflect current patterns of intake were also identified in some regions (Europe and Asia). Given the diversity in data available by region, each jurisdiction will be discussed separately.

Article 27 of Regulation (EU) 1333/2008 requires that all EU Member States monitor population food additive intake [77]. This is likely the reason that the most detailed and up-to-date assessments globally have been conducted in Europe, following a standardized approach, recognized by the European Commission [7], with some studies incorporating further refinements, such as presence probability/occurrence data and market share information. The available assessments have, for the most-part, been conducted using nationally representative samples; data is available for at-risk consumers, such as children, diabetics and brand loyal individuals, as well as children with special dietary requirements (consumers of FSMPs). Most of the European studies identified included an uncertainty analysis in the publication, in line with EFSA recommendations [11]. The results of the European assessments generally do not indicate a concern among average or heavy-level healthy consumers of all ages in Europe under the typical conditions of use for all major low-/no-calorie sweeteners (i.e., Tier 3). More recent studies have generally indicated a reduction in intakes in various EU countries, although differences in design mean that comparisons between studies are limited and must be interpreted with caution. The only instances where intakes were noted to reach or exceed the ADI at Tier 3 were among young children with special dietary requirements (PKU and diabetes) for acesulfame-K, cyclamate and steviol glycosides. In both studies, limitations were identified by the study authors with regard to the following findings: consumption of low-/no-calorie sweeteners in PKU children was modeled using consumption data for healthy children [65,67], and the exceedence of the ADI observed in diabetic children was based on a small sample population (*n* = 9) [63]. While significant limitations were noted, these findings suggest that young children with specific dietary requirements may potentially exceed the ADI for the three sweeteners examined in the EU, indicating that intakes by these groups should be further evaluated. While the ADI is defined as the “amount of a food additive, expressed on a body weight basis that can be ingested daily over a lifetime without appreciable health risk” [94], it is important to remember that the ADI applies to children on the basis that toxicological protocols cover the periods of rapid growth, development and maturation. The possible exceedance of the ADI, on occasion including periods of childhood, has been empasised by JECFA in relation to the large safety factor applied within its derivation. The JECFA indicated that “because...data are extrapolated from lifetime animal studies, the ADI relates to lifetime use and provides a margin of safety large enough for toxicologists not to be concerned about short term use at exposure levels exceeding the ADI, providing the average intake over longer periods does not exceed it” [95]. As such, exceedance of the ADI, identified for children within these two studies, only during a fraction of their lifetime, may not indicate a safety concern, especially as low-/no-calorie sweeteners have a remarkably low acute toxicity potential [70,96,97,98]. The reduction in intake below the ADI at Tier 3 in most studies illustrates the importance of incorporating all available resources to identify the most representative exposure levels in population groups. In studies published prior to 2008, evaluated by Renwick [9], it was noted that the ADI for cyclamate was exceeded in a total of four studies, two conducted in the UK [99,100] and two conducted in Denmark [101,102]. However, more recent data reviewed as part of this analysis, indicated a reduction in estimated daily cyclamate intakes, including among Danish individuals [53]. This is likely due to changes in usage—specifically a reduction in the MPL [103]—and potential associated reformulations. Indeed, several studies noted a very low presence of this sweetener within the Norwegian and Irish markets [52,69].

There was also recent low-/no-calorie sweetener intake data available for Asian consumers, whereby the exposure methods applied varied according to country.

In Japan, up-to-date and regular assessments of sweetener intakes were primarily conducted by the Ministry of Health, Labour and Welfare (MHLW) using the market-based approach. The studies considered all ages of the general population above 1 year, using nationally representative dietary data, combined with analytical data for the foods consumed as part of the dietary surveys. The use of concentration data from foods consumed by the cohort is a strength of this approach as it results in exposure estimates that are specific to the cohort. There were no high percentile estimates examined for the Japanese population, nor investigations of consumption by diabetic individuals, and the analyses generally considered all analytical data (i.e., included foods not identified as containing sweeteners). Nonetheless, the estimated intakes were extremely low in all studies for each of the sweeteners investigated (<1%ADI), with no change in intakes identified between 2009 and 2016, suggesting that there is no safety concern, even for individuals at the upper end of exposure.

The Korean studies available were also up-to-date and examined exposure to an array of low-/no-calorie sweeteners using both deterministic and distributional approaches. The assessments were based on nationally representative dietary surveys, considering all ages of the general population, and current analytical data. One study [34] examined a scenario which was identified by the authors to be representative of consumers who were brand loyal to sweetener-containing foods, using the mean concentration of only positive samples. This is different to the approach established in the EU to assess exposure by brand loyal consumers is based on the maximum use level for the ‘brand loyal category’ (and the mean/median typical reported use level for all other categories) [104]. There was no investigation of diabetic individuals in Korea. Overall, the data indicated no concern for the six sweeteners examined among the average and high-level consumers in the general population. An examination of trends across time in several of the studies indicated that there was a reduction in the mean and heavy-level intakes calculated for acesulfame-K, aspartame, saccharin and sucralose in more recent assessments when compared with older data.

Of the two studies identified in China, only one was based on a nationally representative sample [21]. This assessment indicated that there may be exceedances among high-level (97.5th percentile) consumers of cyclamate-containing foods; however the authors noted that the use of MPLs in broad food categories (i.e., no account of actual use levels or presence) may have led to overestimations in intakes. A separate study [20] used a tiered approach to examine exposure to cyclamate and sodium saccharin from preserved fruits only in a cohort of Chinese college students. The refined assessment, which was based on the MPL and individual-based consumption data, identified no exceedance to either sweetener; however, authors did note an exceedance of the MPL for cyclamate in measured samples, indicating that the actual intakes may be higher than those calculated (up to 21%ADI in this scenario).

The Indian cohort presented information on exposure to four sweeteners (acesulfame-K, aspartame, saccharin and sucralose) in a small cohort of high-consuming individuals [22]. The assessment was based on the MPL or reported use levels for table-top sweeteners. The intakes were examined as means only, which were well below the ADI. Given the cohort examined, i.e., diabetics, overweight individuals and female college studies, the estimated intakes may be representative of high consuming ‘average’ consumers in India.

The variation in the exposure assessment methods by country in Asia mean that it is difficult to make overarching conclusions for this region. A focus on nationally representative data is critical, to allow findings to be extrapolated to the wider population. The majority of studies used actual chemical concentration data, which increases the accuracy of the findings. Information on the presence data or market share information could help to further increase the accuracy of the findings. In general, the exposure analyses in Asia indicated that the sweetener intakes were low and usually below the ADI. However, there were no investigations into dietary intake amongst diabetic individuals in this region who are considered high consumers of low-/no-calorie sweeteners. The exposure profile within this sub-population should be assessed to determine the levels in relation to the ADI.

A number of studies were identified for Latin American, which reported exposure in several South American countries. The available assessments were typically conducted in small samples, which were not nationally representative, considering actual reported use levels (from branded foods). There was no account for presence or market share in the exposure assessment calculations. Nonetheless, the results available, which considered children and diabetic individuals, did not indicate a concern based on current patterns of use for six sweeteners. There were some exceedances of the ADIs for cyclamate and saccharin for children (maximum consumers only) and diabetic individuals, respectively. No assessment of intake over time was possible due to variations in the methods applied. Renwick [9] identified a single Latin American study conducted in Brazil (1990–1991). The ADIs were not exceeded for the evaluated sweeteners (aspartame, cyclamate and saccharine); however high-level consumer-only intakes were not estimated. This aligns with research identified as part of the current review. Future assessments should consider high level intakes and seek to conduct analyses for nationally representative cohorts. There were no studies identified which examined sweetener intake in Brazil or Mexico, where national consumption data are available for analysis, including the Brazilian Pesquisa de Orçamentos Familiares and Mexican ENSANUT [105,106,107,108,109]. These data are a valuable resource for adding to the available body of evidence for this region. Food consumption from the ongoing Latin American Study of Nutrition and Health/Estudio Latinoamericano de Nutrición y Salud (ELANS) will also provide a valuable resource for future assessments [110].

The only available estimates for Australian population groups were those conducted by FSANZ for steviol glycosides and acesulfame-K in response to requests to extend the use of these two sweeteners in foods. It was determined that there is no safety concern with the estimated intakes for these sweeteners based on assessments conducted using the MPLs for the sweeteners, rather than current conditions of use. Occurrence data were not considered in the assessments, however, an assumption for the percent market share uptake of steviol glycoside was utilized for non-brand loyal categories. This is similar to the approach taken by EFSA for refined assessments of food additive intake [104]. The availability of nationally representative data provides a good resource for examining exposure in this region to low-/no-calorie sweeteners for the general population in the future.

Global assessments were identified for two sweeteners, namely, steviol glycosides and cyclamate, which indicated that there was no concern associated with the proposed or permitted conditions of their use in the general population. However, the data included were typically *per capita* estimates from the GEMS/Food dataset and/or poundage data (steviol glycoside), or data which was quite dated (cyclamate assessment), which do not provide detailed data for individual consumers or potential at-risk groups. This intake approach is generally used as an initial screening stage for sweeteners under investigation and may be used to guide more accurate assessments.

North American assessments provided limited data, reporting only the percent consumers for the US NHANES, without specific examination into intakes among the population. The assessments available have not investigated actual daily exposure estimates, although it was observed that the proportion of the population consuming low-/no-calorie sweeteners has increased since 1999 [88,89,90]. Information from the food industry and/or analytical data would be valuable for understanding whether the increase in the percentage of consumers is associated with a change in the actual daily exposure levels by consuming individuals. A total of five North American studies (three conducted in the US and two conducted in Canada) were previously identified by Renwick [9]. The results showed that the estimated intake of sweeteners was not exceeded in any of the studies, including at-risk populations, such as healthy children, diabetic children, and diabetic adults. However, it is important that a more up-to-date assessment is conducted, based on current inclusion levels of low-/no-calorie sweeteners, to determine whether there have been changes in exposure among the (growing) consuming population.

Finally, there were no data available for any African countries. The South African NHANES was published in 2012 [111], which will allow investigation of intakes in this country. In the absence of consumption data for other African countries, screening methods would be valuable to provide top-line estimations of exposure.

The authors note that the literature search and subsequent original language searches were comprehensive; however, some data may not have been identified for some regions due to studies not being in the public domain or searches published in additional languages not considered.

## 5. Conclusions and Recommendations

Overall, the studies conducted to determine the exposures of low-/no-calorie sweeteners since 2008 raise no concerns with respect to exceedance of individual sweetener ADIs among the general population globally. The current data identified also do not suggest a significant shift in exposure over time, with several studies indicating a reduction in intake. While exceedances have been noted for cyclamate, acesulfame-K, steviol glycosides and saccharin in some populations, these are generally only among high-level consumers and/or specific sub-groups of the population, such as diabetics and children with special medical requirements.

The ADI was selected as an appropriate parameter to investigate potential safety concerns for low-/no-calorie sweetener intakes as this is the primary health-based guidance value used by competent authorities globally [112], and also the value used in the available intake studies identified from the literature search. Another area of concern, for which there is a growing body of research, is the potential association between low-/no-calorie sweeteners and health parameters, including weight management and obesity, cardiometabolic health, and diabetes, among other health effects. However, there is no conclusive evidence that high intakes of low-/no-calorie sweeteners are associated with these health conditions [113,114,115,116]. Nonetheless, researchers should continue to monitor the available data and consider all intake information for these additives in light of the most robust and concrete scientific literature.

There are data gaps/limitations in the information available, which vary according to region. Differences in the methods used varied not only within a country but also across regions; therefore, limited comparisons between results can be made. To address these limitations, future research should consider a more standardized approach, which will allow trends over time to be examined–an important consideration in light of changes in product formulations. Furthermore, it is important that the population groups examined are considered representative of the wider cohort for whom they represent, e.g., general population or diabetic individuals, and that the chemical concentration data used is representative of products ingested by the consuming population. While data was available for diabetic individuals, there are other subgroups of the population who may be exposed to higher than average levels of low-/no-calorie sweeteners. One such group are individuals on weight loss diets. There was very limited information (only one sample in India [22]) in regard to intake by this cohort. While this study did not suggest a concern with respect to the ADI for sweeteners examined, additional data in more regions would be necessary to make any conclusions regarding exposure within this subgroup. In terms of gathering detailed food consumption data for a population group, for which none exists, Kroes et al. [12] and EFSA [117] have reviewed the available data collections methods; the EFSA guidance document has made recommendations about dietary recall approaches for different cohorts. In countries where there are nationally representative food consumption data available, these should be combined with the best available concentration data to obtain estimates of exposure to sweeteners. With respect to chemical concentration data, information on the presence of sweeteners (as sweeteners are normally used in blends/mixtures, and technological limitations restrict use within food categories), and data on the market share (ensuring the information is representative of products being consumed) are also very valuable resources for obtaining the most accurate assessment of intakes. They may also help to guide assessments based on evaluations of patterns of use of sweeteners.

Overall, while the robustness of the data can obviously be improved in the future in various locations globally, the data provide a significant level of comfort that there does not appear to be a significant shift in low-/no-calorie sweeteners intake and levels of exposure are generally within the ADI limits for the individual sweeteners. However, it is considered important to continue to monitor potential exposures based upon events such as the recent requirement to reduce the level of sugar intake, to ensure there is no shift in intakes, particularly for high-risk individuals, such as diabetics and children with specific dietary requirements, and to ensure risk management decisions are based upon quality intake analyses.

## Figures and Tables

**Table 1:** Estimated Daily Intakes of Low-/No-Calorie Sweeteners in Asia.

Country, Reference	Population Group Examined (*n*)	Consumer Daily Intake (%ADI) ^1^			Conclusions	Comments/Uncertainty Analysis Findings ^4^
		Sweetener Name	Average ^2^	High Level ^3^		
China, Liu et al., 2012 [20]	Female college students, 18–25 years (*n* = 2044). Cohort of students attending 10 different schools in the Guangdong province	☐Ace-K	-	-	Attention to exposure of sweetener use in preserved fruits should be considered as part of the risk management for these foods.	Female college students were examined as they were considered to be high consumers of preserved fruits (+). Assessment only considered sweetener intake from preserved fruit (−). Default body weight used to examine exposure (+/−). Use of point estimate model or MPL (+).
		☐Aspartame	-	-		
		☒Cyclamate	*6.5*–94.25	12.61–15.99 ^5^		
		☒Saccharin	*8.9*–68.14	17.33–21.99 ^5^		
		☐Steviol	-	-		
		☐Sucralose	-	-		
		☐Thaumatin	-	-		
China, Cao et al., 2016 [21]	All ages, ≥2 years; 2 to 3 years; 4–9 years; 10–17 years; 18–59 years; >60 years (*n* = NR). Participants of China Nutrition and Health Survey (2002)	☐Ace-K	-	-	The sodium cyclamate dietary exposure of whole Chinese population was below the ADI. The sodium cyclamate exposure in high exposure individuals (97.5th percentile) should be monitored.	Nationally representative food consumption data. The use of the MPL ^7^ (+). Broad food categories (+). Food consumption data utilised was gathered 10 years prior to the publication-consumption patterns may have changed (+/−).
		☐Aspartame	-	-		
		☒Cyclamate ^6^	*11.6–33.8*	78.08–**246.32**		
		☐Saccharin	-	-		
		☐Steviol	-	-		
		☐Sucralose	-	-		
		☐Thaumatin	-	-		
India, Singhal and Mathur, 2008 [22]	Cohort of assumed heavy consumers—diabetics (*n* = 72), OW individuals (*n* = 39), and female college students (from three colleges; *n* = 47) ^8^; Age range NR	☒Ace-K	*2.8–5.2*	ND	Sweetener intake among diabetics, overweight individuals, and college girls in Delhi is below the ADI.	Focused on only sweetener consumers among a cohort of high consuming individuals (+). Small sample size (+/−) Intakes presented for regular consumers only ^8^ (+). No assessment of high-level intakes (−−). Use of MPL (+).
		☒Aspartame	*2.1–4.8*			
		☐Cyclamate	-			
		☒Saccharin	*16.7–22.4*			
		☐Steviol	-			
		☒Sucralose	*0.5–2.1*			
		☐Thaumatin	-			
Japan, Sadamasu et al., 2009 [23]	Participants of Tokyo Metropolitan Health and Nutrition Survey (2004) (age range ^9^ and sample size NR)	☒Ace-K	0.0029	ND	The estimated intakes were below the ADIs, and this indicated no health concern.	No estimate of high level intakes (−−). Default body weight values used to examine as a %ADI (+/−). Analytical data included zero values (−).
		☒Aspartame	0.0088			
		☐Cyclamate	-			
		☒Saccharin	0			
		☐Steviol	-			
		☐Sucralose	-			
		☐Thaumatin	-			
Japan, MHLW, 2010 [24]	Children, aged 1–6 years (*n* = 2123). Participants of National Nurtition Survey (2001–2002) and National Health and Nutrition Survey (2003)	☒Ace-K	0.23	ND	The estimated intakes for children were below the ADIs, and this indicated no health concern.	Nationally representative food consumption data. No estimation of high-level intakes (−−). Default body weight values used to examine as a %ADI (+/−). Analytical data included zero values (−).
		☐Aspartame	-			
		☐Cyclamate	-			
		☒Saccharin	0.07			
		☐Steviol	-			
		☐Sucralose	-			
		☐Thaumatin	-			
Japan, Kawasaki et al., 2011 [25]	Adults, ≥20 years (*n* = 28,062). Participants of National Nurtition Survey (2001–2002) and National Health and Nutrition Survey (2003)	☒Ace-K	0.08	ND	The estimated daily intake of food additives were far below the ADI. The results suggest that the daily intakes of food additives in the consumption of daily foodstuffs are within safe ranges in Japan.	Nationally representative food consumption data. No estimation of high-level intakes (−−). Default body weight values used to examine as a %ADI (+/−).
		☒Aspartame	0.018			
		☐Cyclamate	-			
		☒Saccharin	0.06			
		☐Steviol	-			
		☒Sucralose	0.018			
		☐Thaumatin	-			
Japan, MHLW, 2011 [26]	Adults, ≥20 years (*n* = 21,890). Participants of National Health and Nutrition Survey (2004–2006)	☒Ace-K	0.35	ND	The estimated intakes for adults were below the ADIs, which indicated no health concern.	Nationally representative food consumption data. No estimation of heavy level intakes (−−). Default body weight values used to examine as a %ADI (+/−). Analytical data included zero values (−).
		☐Aspartame	-			
		☐Cyclamate	-			
		☒Saccharin	0.13			
		☐Steviol	-			
		☐Sucralose	-			
		☐Thaumatin	-			
Japan, MHLW, 2012 [27]	Ages 1–6 years; 7–14 years; 15–19 years; ≥20 years; All ages ≥1 years (*n* = 4510). Participants of Special Survey of the Frequency and Intake of Food Consumption (2010)	☒Ace-K	0.210–0.447	ND	The estimated intakes for all ages were below the ADIs, which indicated no health concern.	Nationally representative food consumption data. No estimation of high-level intakes (−−). Default body weight values used to examine as a %ADI (+/−). Analytical data included zero values (−).
		☒Aspartame	0.001–0.004			
		☐Cyclamate	-			
		☒Saccharin	0.076–0.163			
		☒Steviol	0.119–0.259			
		☒Sucralose	0.084–0.186			
		☐Thaumatin	-			
Japan, Sato et al., 2013 [28]	Total population	☒Ace-K	0.82	ND	There was no significant change in the amount of daily intake of the approved additives compared to the past surveys with no additives that exceed the ADI.	No account of intake by consumers only (−−). No estimation by high level consumers (−−). Default body weight values used to examine as a %ADI (+/−).
		☒Aspartame	0.24			
		☐Cyclamate	-			
		☒Saccharin	0–0.58			
		☐Steviol	-			
		☒Sucralose	0.32			
		☐Thaumatin	-			
Japan, Kumai et al., 2015 [29]; MHLW, 2015 [30]	Children 1–6 years (*n* = 227). Participants of Special Survey of the Frequency and Intake of Food Consumption (2010)	☒Ace-K	0.14	ND	The estimated intakes for children were below the ADIs, and this indicated no health concern.	Nationally representative food consumption data. No assessment of high-level intakes (−−). Default body weight used to examine intakes on a %ADI (+/−). Analytical data included zero values (−).
		☐Aspartame	-			
		☐Cyclamate	-			
		☐Saccharin	-			
		☐Steviol	-			
		☒Sucralose	0.15–0.16			
		☐Thaumatin	-			
Japan, MHLW, 2016 [31]	Ages 1–6 years; 7–14 years; 15–19 years; ≥20 years; All ages ≥1 years (*n* = 4510). Participants of Special Survey of the Frequency and Intake of Food Consumption (2010)	☒Ace-K	0.13–0.26	ND	The estimated intakes were below the ADIs, and this indicated no health concern.	Nationally representative food consumption data. No assessment of high-level intakes (−−). Default body weight used to examine intakes on a %ADI (+/−). Analytical data included zero values (−).
		☒Aspartame	0			
		☐Cyclamate	-			
		☒Saccharin	0.03–0.06			
		☒Steviol	0.20–0.47			
		☒Sucralose	0.07–0.15			
		☐Thaumatin	-			
Korea, Choi et al., 2011 [32]	All ages (age range and sample size NR). Participants of KNHANES (2005)	☒Ace-K	4.9	14.6	Sweteeners are safely consumed by the Korean population, inclduing 95th percentile consumers.	Nationally representative food consumption data. Use of analytical data. Intakes summed for individual food groups (+).
		☒Aspartame	4.9	15.8		
		☐Cyclamate	-	-		
		☒Saccharin	7.5	20		
		☐Steviol	-	-		
		☒Sucralose	8.6	23.7		
		☐Thaumatin	-	-		
Korea, Lee et al., 2011 [33]	Children and adolescents (*n* = 6625); 0–6 years; 7–12 years; 13–19 years. Participants of a national dietary survey, Dietary Intake Survey of Infant, Children and Adolescents (2007–2009)	☒Ace-K	*1.49*	*2.66*	Sweetener intake from snacks targeted towards children is low among Korean children.	Nationally representative food consumption data. Use of analytical data.
		☒Aspartame	*0.72*	*1.81*		
		☐Cyclamate	-	-		
		☒Saccharin	*0.08*	*0.15*		
		☐Steviol	-	-		
		☒Sucralose	*0.24*	*0.45*		
		☐Thaumatin	-	-		
Korea, Ha et al., 2013 [34]	Total population	☒Ace-K	***293.3***	ND	Screening tool indicated that a more refined approach was required to investigate the actual EDIs of sweeteners.	Budget method is a screening technique. Default values used for food and beverage consumption, use of MPL (+++).
		☐Aspartame	-			
		☐Cyclamate	-			
		☐Saccharin	-			
		☐Steviol	-			
		☒Sucralose	***173.3***			
		☐Thaumatin	-			
	All ages, 1 to >65 years (*n* = 8081). Participants of KNHANES who consumed intense sweeteners during the 24-h recall (2009)	☒Ace-K	*4.0–23.3*	*14.0–66.0*	The upper 95th percentile of consumers (i.e., Scenario B; only positive samples included) are at risk of exceeding the ADI for sucralose. No exceedence for ace-K.	Nationally representative food consumption data. Use of analytical data. ‘Scenario B‘ considered the values for only the positive mean samples (detection rate for the sweeteners ranged from 2 to 63%) (+).
		☐Aspartame	-	-		
		☐Cyclamate	-	-		
		☐Saccharin	-	-		
		☐Steviol	-	-		
		☒Sucralose	*7.3–44.7*	*22.7–**118.0***		
		☐Thaumatin	-	-		
Korea, Ha et al., 2013 [35]	All ages, 1 to >65 years (*n* = 8081). Participants of KNHANES who consumed intense sweeteners during the 24-h recall (2009)	☐Ace-K	-	-	EDIs of all sweeteners for all age groups and even for the 95th percentile consumers were lower than their ADIs, even under Scenario B	Nationally representative food consumption data. Use of analytical data. ‘Scenario B‘ considered the values for only the positive mean samples (detection rate for the sweeteners ranged from 4 to 100% (+).
		☒Aspartame	*4.2–20.0*	*14.1–49.5*		
		☐Cyclamate	-	-		
		☒Saccharin	*7.2–24.8*	*16.0–50.4*		
		☒Steviol	*6.1–14.3*	*19.8–35.2*		
		☐Sucralose	-	-		
		☐Thaumatin	-	-		
Korea, Suh and Choi, 2013 [36]	All ages (sample size NR); 1–2 years; 3–6 years; 7–12 years; 13–19 years; 20–29 years; 30–39 years; 40–49 years; 50–64 years; >65 years. Participants of KNHANES (2010)	☐Ace-K	-	-	Saccharin and sucralose are safely consumed among the general Korean population.	Nationally representative food consumption data. Use of analytical data. Intakes summed for individual food groups (+).
		☐Aspartame	-	-		
		☐Cyclamate	-	-		
		☒Saccharin ^10^	1.181	5.29		
		☐Steviol	-	-		
		☒Sucralose ^10^	0.551	15.66		
		☐Thaumatin	-	-		
Korea, Kim et al., 2014 [37]	Children and adolescents, aged 1–19 years (*n* = 6082). Participants of KNHANES (2007–2009)	☒Ace-K	0.07–0.22	0.00–0.80	No issue with sweetener intake from non-alcoholic beverages among Korean children and adolsecents.	Nationally representative food consumption data. Use of analytical data. Assessment considered beverages only (−).
		☒Aspartame	0.05–1.32	0.00–4.52		
		☐Cyclamate	-	-		
		☐Saccharin	-	-		
		☐Steviol	-	-		
		☒Sucralose	0.27–1.48	0.00–5.06		
		☐Thaumatin	-	-		
Korea, Suh et al., 2014 [38]	All ages (sample size NR); 1–2 years; 3–6 years; 7–12 years; 13–19 years; 20–29 years; 30–39 years; 40–49 years; 50–64 years; >65 years. Participants of KNHANES (2010)	☒Ace-K ^10^	0.091	5.08	Ace-k and aspartame are safely consumed among the general Korean population.	Aspartame and ace-K were studied as there were reported to be the most frequently utilized artificial sweeteners in Korea. Nationally representative food consumption data. Use of analytical data. Intakes summed for individual food groups (+).
		☒Aspartame ^10^	0.151	6.28		
		☐Cyclamate	-	-		
		☐Saccharin	-	-		
		☐Steviol	-	-		
		☐Sucralose	-	-		
		☐Thaumatin	-	-		
Korea, Lee et al., 2017 [39]	All ages (*n* = 34,706). Participants of KNHANES (2010–2014)	☒Ace-K	1.7	6.7	High level consumers among the general population of sweeteners are not at risk; Recommendation to examine intakes of children separately.	Nationally representative food consumption data. Use of analytical data. Assessment considered the values for only the positive mean samples (detection rate for the sweeteners ranged from 2 to 91%) (+).
		☒Aspartame	0.9	3.8		
		☐Cyclamate	-	-		
		☒Saccharin	3.6	12.8		
		☐Steviol	-	-		
		☒Sucralose	2.2	7.3		
		☐Thaumatin	-	-		
Korea, Kim et al., 2017 [40]	All ages; <2 years; 3–6 years; 7–12 years; 13–19 years; 20–64 years; >65 years (*n* = 20,788). Participants of KNHANES (2010–2013)	☒Ace-K ^11^	*1.7–5.2*	*6.3–20.1*	No issue with sweetener intake among the general Korean population.	Nationally representative food consumption data. Use of analytical data. Assessment considered the values for only the positive mean samples (detection rate 16–23%) (+).
		☒Aspartame	*0.4–3.2*	*1.7–10.0*		
		☐Cyclamate	-	-		
		☒Saccharin	*2.8–5.4*	*10.6–18.4*		
		☐Steviol	-	-		
		☒Sucralose	*1.3–3.9*	*4.1–12.5*		
		☐Thaumatin	-	-		
	Total population	☐Ace-K	-	ND	No issue with sweetener intake among the general Korean population.	Poundage method does not account for actual intakes by consuming individuals (−−). No calculation of high level consumers (−−). Default body weight used to examine intakes on a %ADI (+/−).
		☐Aspartame	-			
		☐Cyclamate	-			
		☐Saccharin	-			
		☒Steviol ^12^	4.3			
		☐Sucralose	-			
		☒Thaumatin	ADI NS			

Ace-K = acesulfame-K; ADI = acceptable daily intake; EDI = estimated daily intake; h = hours; KNHANES = Korean National Health and Nutrition Survey; MPL = maximum permitted level; *n* = sample size; NS = not specified; NR = not reported; ND: not determined; OW: overweight. ^1^ Results are based on ADIs derived by JECFA, unless otherwise stated; figures are **bolded** when exceed the ADI; figures are *italicized* when results as %ADI calculated based on data reported in the publication (as mg/kg bw/day); ^2^ Average intakes are presented as the mean consumption level; median intakes are presented if mean was not available; ^3^ High level consumers are defined as P95 unless otherwise indicated; ^4^ Comments and uncertainty analysis findings based on information reported by study authors, or data identified from the study. Sources of under- or over-estimation identified by (−)/(+); +, ++, +++ are the uncertainties likely to cause small, medium or large overestimates of exposure; −, −− are the uncertainties likely to cause small or medium underestimates of exposure. Information may not be comprehensive for all models available; ^5^ Figure presented for 95% confidence interval. These values are lower than the average intake estimates as only average values were calculated for the deterministic data, while the 95% confidence interval was calculated only for the simple distribution model; ^6^ Results are based on ADIs derived by EFSA (7 mg/kg bw/day). High level consumers are defined as the 97.5th percentile; ^7^ Values are reported as maximum residue limit (MRL) in the publication; however, the MPL is the standard terminology for food additives, and used herein; ^8^ The number of individuals (*n*) and results are presented for ‘regular consumers’ only (defined as individuals consuming artificial sweeteners ≥1 a week); intakes by ‘occasional consumers’ (defined as individuals consuming once a fortnight or less) were not examined; ^9^ The study does not specifically state the age group investigated; however ADI intakes were calculated using a default body weight of 50 kg, which aligns with the default value utilized for adults in other Japanese studies; ^10^ Mean intakes are reported for the total population (consumers and non-consumers); ^11^ Results are calculated based on ADI derived by EFSA (9 mg/kg bw/day), as this was the ADI reported by the authors; ^12^ Based on the reported estimated intakes of stevioside, applying a conversion factor of 0.4.

**Table 2:** Estimated Daily Intakes of Low-/No-Calorie Sweeteners in Australia/New Zealand.

Country, Reference	Population Group Examined (*n*)	Consumer Daily Intake (%ADI) ^1^			Conclusions	Comments/Uncertainty Analysis Findings ^4^
		Sweetener Name	Average ^2^	High Level ^3^		
Australia and New Zealand, FSANZ, 2010, 2011 [43,44,45]	Australian children, 2–16 years (*n* = 4487); general Australian population, ≥2 years (*n* = 13,858); New Zealand children, 5–14 years (*n* = 3275); general New Zealand population, ≥15 years (*n* = 4636)	☐Ace-K	-	-	The 30% market uptake scenario (non-brand loyal), resulted in exposure of up to 60% of the ADI for average and 90th percentile consumers for all population groups assessed, including children. In the brand loyal scenario, exposure was exceeded at the 90th percentile of exposure (up to 110% for children 2–6 years); however, given the conservative nature of these assessments, there is no issue with the proposed increases in MPLs.	The 30% market share scenario and subsequent ‘brand loyal’ consumer scenarios are based on very conservative assumptions that are likely to lead to a considerable overestimation of dietary exposure. Nationally representative food consumption data. Used MPL (with/without market share) (+).
		☐Aspartame	-	-		
		☐Cyclamate	-	-		
		☐Saccharin	-	-		
		☒Steviol	10–55	20–**110**		
		☐Sucralose	-	-		
		☐Thaumatin	-	-		
Australia and New Zealand, FSANZ, 2015 [46,47,48]	Australian children, 2–6 years (*n* = 779) and 7–11 years (*n* = 802); general Australian population, ≥2 years (*n* = 12,153) and ≥12 years (*n* = 10,572); New Zealand children, 5–14 years (*n* = 3275); general New Zealand population, ≥15 years (*n* = 4721)	☒Ace-K	5–7	9–15	No issue with the proposed increases in MPL for chewing gum. Intakes could only be examined by individuals ≥12 years in Australia and ≥15 years in New Zealand, as there was no data from the total diet for these age groups. Still, <1% of younger indivdiuals consumed intensely sweetened chewing gum, which is assumed to have a negligeable effect on ace-K intake from the total diet.	Nationally representative food consumption data. Did not consider presence data (+). All intensely sweetened chewing gum was assumed to contain ace-K at the proposed maximum level; in reality ace-K may be used at lower levels in combination with other sweeteners (+). Cumulative estimates were based on consumption data and concentration data from different time periods (+/−). Exposure from intensely sweetened chewing gum would be counted twice, as it was also included in the 2004 sweetener survey (++).
		☐Aspartame	-	-		
		☐Cyclamate	-	-		
		☐Saccharin	-	-		
		☐Steviol	-	-		
		☐Sucralose	-	-		
		☐Thaumatin	-	-		

Ace-K = acesulfame-K; ADI = acceptable daily intake; FSANZ = Food Standards Australia New Zealand; JECFA = Joint FAO/WHO Expert Committee on Food Additives; MPL = maximum permitted level; *n* = sample size. ^1^ Results are based on ADIs derived by JECFA; figures are **bolded** when the ADI is exceeded; ^2^ Average intakes are presented as the mean consumption level; ^3^ High level consumers are defined as the 90th percentile; ^4^ Comments and uncertainty analysis findings are based on information reported by study authors, or data identified from the study. Sources of under- or over-estimation are identified by (−)/(+); +, ++, +++ are the uncertainties likely to cause small, medium or large overestimates of exposure; −, −− are the uncertainties likely to cause small or medium underestimates of exposure. Information may not be comprehensive for all models available.

**Table 3:** Estimated Daily Intakes of Low-/No-Calorie Sweeteners in Europe.

Country, Reference	Population Group Examined (*n*)	Consumer Daily Intake (%ADI) ^1^			Conclusions	Comments/Uncertainty Analysis Findings ^4^
		Sweetener Name	Average ^2^	High Level ^3^		
Portugal, Lino et al., 2008 [54]	Adolescents, 13–15 years (*n* = 65) Cohort of students attending a public high school in Coimbra (2006-2007)	☒Ace-K	0.6–8.0	ND	Low risk of excessive intake of aspartame and acesulfame-K among Portugese adolescents.	No assessment of high-level intakes (−−). Default body weight used to examine intakes as a %ADI (+/−). Intakes calculated for individual beverages (not cumulative from all beverages or from total diet) (−). Consumption data based on average annual estimates (−).
		☒Aspartame	0.07–2.9			
		☐Cyclamate	-			
		☐Saccharin	-			
		☐Steviol	-			
		☐Sucralose	-			
		☐Thaumatin	-			
Denmark, Leth et al., 2008 [53]	Total population 1-80 years (*n* = 3098); young children 1–3 years (*n* = 278); children 4–6 years (*n* = 366); children 7–10 years (*n* = 376). Participants of Danish Dietary Survey (1995)	☒Ace-K	*0.13–4.00*	*2.3–32.2 ^5^*	The estimated intake of Ace-k, aspartame and saccharin were well below their respective ADIs, even at the maximum level of intake. Estimated intake of cyclamate was well below the ADI for the average and 90th percentile intake estimate, only exceedence was at the 99th percentile for 1–3 year olds (105.29%ADI). No significant difference between average and high-level intakes–linked to the use of a mix of sweeteners in soft drinks without carbon dioxide.	Nationally representative food consumption data. Consumption patterns of non-alcoholic beverages may have changed over the past 20 years (+/−). Only beverages considered (−). High level consumer estimates considered at the 99th percentile (+).
		☒Aspartame	*0.08–1.25*	*1.30–10.70* ^5^		
		☒Cyclamate	*0.57–13.00*	*11.14–**105.29*** ^5^		
		☒Saccharin	*0.20–3.20*	*2.00–26.00* ^5^		
		☐Steviol	-	-		
		☐Sucralose	-	-		
		☐Thaumatin	-	-		
Norway, Husøy et al., 2008 [52]	Participants from 4 national dietary surveys. Young children, 1 year and 2 years from Spedkost (*n* = 1204) and Småbarnskost (*n* = 1720), repsectively; Children, 4-, 9-, and 13 years (*n* = 2215) from Ungkost; Adults, 16–79 years (*n* = 2672) from Norkost food survey (1997); and Adults 16–80 years (*n* = 1375) from Omnibus survey (1997)	☒Ace-K	*3.3–18.9*	*1.6–72.2*	Few beverages in Norway contain cyclamate or saccharin–intake was negligible for all ages. The intake of Ace-K in small children approached the ADI, and contribution from other food sources might lead to an exceedence of ADI. Intakes of aspartame were well below the ADI for any age group. Although only intake from beverages were examined for children, it is unlikely that the contribution from foods would increase the estimates above the ADI.	Nationally representative food consumption data. Use of use levels and analytical data, combined with market share. No analysis of intake from food and beverages combined ^6^ (−).
		☒Aspartame	*2.5–5.8*	*2.0–21.0*		
		☒Cyclamate	NR	*41.46*		
		☒Saccharin	NR	*3.86*		
		☐Steviol	-	-		
		☐Sucralose	-	-		
		☐Thaumatin	-			
France, Bemrah et al., 2008 [51]	Participants of national dietary survey (INCA1, 1998–1999). All ages (*n* = 3033); children and young teenagers, 3-14 years (1018); adults, ≥15 years (*n* = 1985)	☐Ace-K	-	-	No issue with intake of cyclamate among French children and adults.	Nationally representative food consumption data. Intakes determined based on the mean concentration for each food category, although the additive concentration varied among different brands (+/−).
		☐Aspartame	-	-		
		☒Cyclamate	0.1–0.4	0.9–5.2 ^7^		
		☐Saccharin	-	-		
		☐Steviol	-	-		
		☐Sucralose	-	-		
		☐Thaumatin	-	-		
Austria, Mischek, 2010 [56]	National consumption data available from Australian Nutrition Report 2003 for Preschool children (3–6 years) and adults (*n* = 151 and 2581, respectively)	☒Ace-K	5.0–7.9	11.8–25.1	The estimated daily intakes for all studied sweeteners were well below the respective ADIs. The consumption of beverages containing intense sweeteners does not pose a health risk to consumers	Nationally representative food consumption data. Default body weights were used for preschool children and adults (+/−). Did not consider total diet (−).
		☒Aspartame	0.7–1.1	1.7–3.6		
		☒Cyclamate	6.3–10.4	8.0–16.7		
		☒Saccharin	3.2–5.6	14.9–32.4		
		☐Steviol	-	-		
		☐Sucralose	-	-		
		☐Thaumatin	-	-		
Portugal, Lino and Pena, 2010 [55]	Total population	☐Ace-K	-	ND	No issue with intake of saccharin among the general Portuguese population.	No assessment of high-level intakes (−−). Default body weight used to examine intakes as a %ADI (+/−). Intakes calculated for individual beverages (not cumulative from all beverages or from total diet) (−). Consumption data based on average annual estimates (−).
		☐Aspartame	-			
		☐Cyclamate	-			
		☒Saccharin	0.00–1.28			
		☐Steviol	-			
		☐Sucralose	-			
		☐Thaumatin	-			
The Netherlands, Hendriksen et al., 2011 [57]	Young adults, 19–30 years (*n* = 750). Participants of DNFCS 2007-2010	☒Ace-K	*2.2–5.0*	*27.2–29.4*	No issue with sweetener intake among young, healthy Dutch adults.	Nationally representative food consumption data. Did not account for intake from sources other than carbonated soft drinks (−).
		☒Aspartame	*0.4–1.0*	*5.1–5.6*		
		☒Cyclamate	*0–5.3*	*29.0–36.7*		
		☒Saccharin	*0*	*3.4–3.6*		
		☐Steviol	-	-		
		☐Sucralose	-	-		
		☐Thaumatin	-	-		
Belgium, Huvaere et al., 2012 [58]	Adults, aged ≥15 years (*n* = 3083), including diabetics (*n* = 428). Participants of the Belgian Food Consumption Survey (dating from 2004)	☒Ace-K	*5.9–11.2*	*13.4–41.7*	Belgian adults are not at risk of exceeding the ADI for examined sweeteners, including diabetics, considering both the MPL and analytical data for these sweeteners.	Nationally representative food consumption data. The food label survey indicated that recently approved sweeteners—neohesperidine dihydrochalcone, thaumatin and neotame—were not found on the local market; as such, intakes were not assessed. No intake assessment in children; however, the ADI is based on life-long exposure, exceeding the the ADI in childhood will possibly be compensated by a low intake in adulthood (as shown by this work) and thus will not compromise conclusions on the safety of the intake of sweeteners in Belgium.
		☒Aspartame	*1.5–4.9*	*3.4–16.9*		
		☒Cyclamate	*3.3–6.3*	*12.7–29.4*		
		☒Saccharin	*3.0–6.8*	*7.4–22.8*		
		☐Steviol	-	-		
		☒Sucralose	*2.8–5.6*	*5.5–20.5*		
		☐Thaumatin	-	-		
Portugal, Diogo et al., 2013 [59]	Total population	☒Ace-K	0.0–0.7	ND	The Portuguese population is not at risk of exceeding the established. ADIs for the investigated sweeteners	No assessment of high-level intakes (−−). Default body weight used to examine intakes on a %ADI (+/−). Intakes were calculated for individual beverages (not cumulative from all beverages or from total diet) (−). Consumption data based on average annual estimates (−).
		☒Aspartame	0.0–0.08			
		☐Cyclamate	-			
		☒Saccharin	0.0–0.9			
		☐Steviol	-			
		☐Sucralose	-			
		☐Thaumatin	-			
France, Italy, UK, Ireland, Vin et al., 2013 [60]	France-Participants of the INCA 2 (2005–2007) aged 3–79 years (*n* = 4079). Italy-Participants of the INRAN-SCAI (2005–2006), aged 1 mth to 97 years (*n* = 3323). UK-Participants of the UK NDNS (1992–2001), aged 1.5 to >65 years (*n* = 6787). Ireland, Participants of the NSIFCS (1997–1999), NCFS (2003–2004), or NTFS (2005–2006), aged 5–64 years (*n* = 2414)	☒Ace-K	2–69	9–**166** ^7^	High level (97.5th percentile) intake of acesulfame-K exceeded the ADI in children from the UK, France, and Ireland at Tier 2, but not Tier 3, whereas aspartame was below the ADI in all population groups and scenarios. The use of a specific codification system and a fitted distribution of “real” concentrations (instead of MPLs) significantly refined the exposure model and therefore reduced the estimated intake.	Nationally representative food consumption data. “undetermined” products (e.g., fruit and vegetables) were assumed to be “canned”and contain the evaluated sweeteners (+). No account for brand loyal individuals in the Tier 3 assessment (−). No inclusion of occurrence data (+).
		☒Aspartame	0–30	3–83 ^7^		
		☐Cyclamate	-	-		
		☐Saccharin	-	-		
		☐Steviol	-	-		
		☐Sucralose	-	-		
		☐Thaumatin	-	-		
17 EU Member States, EFSA, 2013 [70]	Toddlers, 12–35 months; Children, 3–9 years; Adolescents, 10–17 years; Adults, 18–64 years; Elderly, ≥65 years (*n* = NR)	☐Ace-K	-	-	No safety concerns at the current ADI of 40 mg/kg body weight/day.	MPLs used when no use level data was available (+). Assumed that aspartame was always present in foods when permitted (+). Data from industry could be considered to be non-representative due to the comments provided by respondents.
		☒Aspartame	*1.0–40.8*	*3.5–92.3*		
		☐Cyclamate	-	-		
		☐Saccharin	-	-		
		☐Steviols	-	-		
		☐Sucralose	-	-		
		☐Thaumatin	-	-		
Norway, VKM, 2014 [71]	Children, 2 years (*n* = 1674). Participants of Småbarnskost (2006–2007). Young women, 18–29 years (*n* = 143); Young men, 18–29 years (*n* = 138); 30–70 years (*n* = 782); Men 30–70 years (*n* = 724). Participants of Norkost 3 (2010–2011)	☐Ace-K	-	-	High level intakes of cyclamate approached the ADI in young women assumed to be brand loyal consumers (96%). Similarly, high level intakes of steviol glycosides approached the ADI in children when concentration data was based on MPLs (80%). As both scenarios are considered to be conservative, it was concluded that there is no issue with sweetener intakes among the evaluated age groups.	Nationally representative food consumption data. Exposure was estimated based on the mean concentration or highest reported concentration–they did not use actual intake (+). Intakes were not estimated in the general Norwegian population aged 3 to 17 years as survey data for this age group is outdated (2000–2001). Assessment considered only beverages (−).
		☐Aspartame	-	-		
		☒Cyclamate	8.71–28.57	16.29–96.14		
		☒Saccharin	3.00–10.80	5.80–36.60		
		☒Steviols	2.25–23.25	6.25–79.50		
		☐Sucralose	-	-		
		☐Thaumatin	-	-		
Norway, VKM, 2014 [72]	Children, 2 years (*n* = 1674). Participants of Småbarnskost (2007). Young women, 18–29 years (*n* = 143); Young men, 18–29 years (*n* = 138); 30–70 years (*n* = 782); Men 30–70 years (*n* = 724). Participants of Norkost 3 (2010–2011)	☒Ace-K	4.00–18.56	10.56–59.33	No issue with sweetener intake among the evalauted age groups.	Nationally representative food consumption data. Exposure was estimated based on the mean concentration or highest reported concentration–they did not use actual intake (+). Intakes were not estimated in the general Norwegian population aged 3 to 17 years as survey data for this age group is outdated (2000–2001). Assessment considered only beverages (−).
		☒Aspartame	3.75–9.63	10.80–28.63		
		☐Cyclamate	-	-		
		☐Saccharin	-	-		
		☐Steviols	-	-		
		☒Sucralose	3.67–12.20	9.67–36.73		
		☐Thaumatin	-	-		
France, Mancini et al., 2015 [61]	Children,1–4 months (*n* = 124); 5–6 months (127); 7–12 months (*n* = 195); 13–36 months (*n* = 259). Participants of the BEBE-SFAE dietary survey (2005)	☐Ace-K	-	-	Aspartame exposure in the French population aged 0 to 3 years is far below the ADI.	Nationally representative food consumption data. Use of MPL (+). No inclusion of natural sources or dietary supplements in the assessment (−).
		☒Aspartame	*0.5–35.3* ^8^	*0.0–59.8* ^8,9^		
		☐Cyclamate	-	-		
		☐Saccharin	-	-		
		☐Steviol	-	-		
		☐Sucralose	-	-		
		☐Thaumatin	-	-		
Belgium, Van Loco et al., 2015 [62]	Toddlers, children, adolescents, adults, the elderly (FAIM V1.1) (*n* = NR)	☒Ace-K	*22.22*	*73.33*	Although intakes exceeded the ADI when caluclated using the FAIM template, there is no issue in the Belgian adult population based on Tier 2 exposure estimates derived by Huvaere et al. [58].	Values derived from FAIM tool should be interpreeted with caution due to extreme overestimation (++). Assessment used MPLs (+).
		☒Aspartame	*17.25*	*36.75*		
		☒Cyclamate	*0*	*65.71*		
		☒Saccharin	***7488***	***7514***		
		☐Steviols	-	-		
		☒Sucralose	***9837.33***	***9863.33***		
		☐Thaumatin	-	-		
17 EU Member States, EFSA, 2015 [73]	Toddlers, 12–35 months; Children, 3–9 years; Adolescents, 10–17 years; Adults, 18–64 years; Elderly, ≥65 years	☐Ace-K	-	-	Mean and high level exposure estimates are below the ADI, with the exception of toddlers (in one country) at the upper rage of high level exposure (107.5%ADI). There were negligible change in intakes compared to previous exposure assessment conducted by EFSA in 2014 [80].	Assessment was based on the current and proposed MPLs, with no occurrence (++). Inability to match FoodEx categories resulted in inclusion/exclusion of some categories (+/−).
		☐Aspartame	-	-		
		☐Cyclamate	-	-		
		☐Saccharin	-	-		
		☒Steviols	*2.5–60.0*	*10.0–**107.5***		
		☐Sucralose	-	-		
		☐Thaumatin	-	-		
17 EU Member States, EFSA, 2015 [74]	Toddlers, 12–35 months; Children, 3–9 years; Adolescents, 10–17 years; Adults, 18–64 years; Elderly, ≥65 years	☐Ace-K	-	-	No issue as margin of safety is >1000 at the highest estimated exposure level (1.10 mg/kg bw/day in adults).	Use of summary statistics (FAIM) and MPL at the 100% presence level (++). Use in flavourings not included (−).
		☐Aspartame	-	-		
		☐Cyclamate	-	-		
		☐Saccharin	-	-		
		☐Steviols	-	-		
		☐Sucralose	-	-		
		☒Thaumatin	ADI NS	ADI NS		
Belgium, Dewinter et al., 2016 [63]	Children and adolescents with T1D (*n* = 103); 4–6 years (*n* = 9); 7–12 years (*n* = 35); 13–18 years (*n* = 59). Cohort of T1D patients from the Pediatrics Department of the University Hospitals Leuven (2014)	☒Ace-K	*14.0–56.3* ^8^	*58.0–**211.9***	No exceedence of the ADI at the mean intake for MPL or maximum use levels. Acesulfame-K, cyclamate, and steviol glycoside ADIs were exceeded at Tier 3 by high level consumers (95th percentile) aged 4–6 years. No exceedences were identified among older indivdiuals.	Low number of participants aged 4–6 years (*n* = 9). The individuals surveyed were not representative of the Belgian population as subjects were recruited from a single hospital. Use of MPL for steviol glycosides (due to lack of analytical data) (+).
		☒Aspartame	*1.7–20.5* ^8^	*6.8–79.1*		
		☒Cyclamate	*20.6–60.4* ^8^	*71.3–**216.7***		
		☒Saccharin	*9.0–26.8* ^8^	*37.2–**100.6***		
		☒Steviol	*15.8–33.0* ^8^	*63.5–**118.8***		
		☒Sucralose	*7.0–24.9* ^8^	*32.5–99.1*		
		☐Thaumatin	-	-		
Ireland, Martyn et al., 2016 [64]	Toddlers and Children ages 1–4 years (*n* = 500). Participants of the National Preschool Nutrition Survey (2011–2012)	☒Ace-K	6–31	23–**118**	No issue with sweeteners among this age group based on refined Tier 3 assessment.	Nationally representative food consumption data.Chemical concentration data not linked to consumption data (+/−). MPL used where sweetener concentration data was not available, assessments used LOR for left-censored data and assumed the presence probability where data were missing (+). Tier 3 did not account for brand loyal consumers (+/−). No account for market share (+/−).
		☒Aspartame	2–13	7–46		
		☐Cyclamate	-	-		
		☒Saccharin	4–14	15–50		
		☐Steviol	-	-		
		☒Sucralose	4–17	13–61		
		☐Thaumatin	-	-		
Ireland, O’Sullivan et al., 2016 [65]	Particpants of the Irish National Preschool Nutrition Survey (NPNS; 2010–2011), aged 1–3 years (*n* = 376), used as a surrogate for children with PKU and CMPA	☒Ace-K	66.5–98.9	153.6–**463.3**	Sweetener intake is greater in young children with PKU and CMPA compared to young healthy children; however, exposure to artificial sweeteners from the total diet does not necessarily exceed the ADI.	Absence of actual food consumption data for these patients required modelling (+/−). Different approaches across the EU for prescribing protein (+/−). Results were also presented using the FAIM template for other EU population groups; however this was identified by the authors to not be a suitable dataset, and results were presented only for comparison to Scenario 1 results–as such, they are not presented here.
		☒Aspartame	21.0–68.0	46.5–**149.8**		
		☐Cyclamate	-	-		
		☐Saccharin	-	-		
		☐Steviol	-	-		
		☒Sucralose	16.8–89.3	38.9–**182.0**		
		☐Thaumatin	-	-		
Assessment from FSMPs in Young Children, EFSA, 2016 [75]	Young children, 1–3 years	☐Ace-K	-	ND	No issue with use of sucralose, as proposed.	No account of sweetener intake from food sources other than FSMPs (−). Assumptions required regarding food consumption (+/−).
		☐Aspartame	-			
		☐Cyclamate	-			
		☐Saccharin	-			
		☐Steviols	-			
		☒Sucralose	*19.3–80.7*			
		☐Thaumatin	-			
Assessment from FSMPs in Young Children, EFSA, 2016 [76]	Young children, 1–3 years	☒Ace-K	*45.6–**204.4***	ND	No issue for the use of up to 9 mg ace-K/g protein to provide 10 g protein in this cohort. ADIs were exceeded in other scenarios examined (higher intake of protein from sweetened product).	No account of sweetener intake from food sources other than FSMPs (−). Assumptions required regarding food consumption (+/−).
		☐Aspartame	-			
		☐Cyclamate	-			
		☐Saccharin	-			
		☐Steviols	-			
		☐Sucralose	-			
		☐Thaumatin	-			
Ireland, Buffini et al., 2017 [69]	Adults, 18–90 years (*n* = 1413), Participants of the National Adult Nutrition Survey (2011)	☒Ace-K	*1.22–7.33*	13.10–59.25 ^10^	Intakes for each of the six sweeteners were all below their relevant ADI levels according to crude and refined exposure assessments.	Use of MPLs for missing data in refined assessments (+). Concentration data: some categories not analysed/no repeat analysis (+/−).Tier 3 did not account for brand loyal consumers (+/−). Nationally representative food consumption data.
		☒Aspartame	*1.65–2.60*	17.78–21.62 ^10^		
		☒Cyclamate	*2.43–5.14*	20.61–49.30 ^10^		
		☒Saccharin	*0.80–4.60*	8.59–41.19 ^10^		
		☒Steviol	*0.50–4.00*	2.52–22.05 ^10^		
		☒Sucralose	*0.40–3.73*	4.54–21.54 ^10^		
		☐Thaumatin				
Italy, Le Donne et al., 2017 [66]	All ages, ≥3 years (*n* = 3270). Participants of the INRAN-SCAI (2005–2006)	☒Ace-K	1.1–7	5–27	No issue with sweetener intake among the general Italian population.	Nationally representative food consumption data. Market share data was not specific to sugar-free or sugar-reduced products (+/−). Only four steviol glycosides were analyzed; therefore, it is possible that intake of steviol glycosides was underestimated (−).
		☒Aspartame	0.5–2	0.1–10		
		☒Cyclamate	3–4	13–16		
		☒Saccharin	0.3–3	1–11		
		☒Steviol	0.1–4	1–15		
		☒Sucralose	0.2–3	1–12		
		☒Thaumatin	ADI NS	ADI NS		
Ireland, O’Sullivan et al., 2017 [67]	Young healthy children, 1.5–3 years (*n* = 2096), used as a surrogate for children with PKU. Participants of the UK NDNS (1992–1993; 2008–2010; 2011–2012)	☒Ace-K	35.9–**111.9**	98.9–**213.4**	The estimated intake of acesulfame-K in young children with PKU has decreased since the use of sucralose in FSMP products, reducing the risk of exceeding the ADI	Absence of actual food consumption data for these patients required modelling (+/−). Different approaches across the EU for prescribing protein (+/−).
		☐Aspartame	-	-		
		☐Cyclamate	-	-		
		☐Saccharin	-	-		
		☐Steviol	-	-		
		☒Sucralose	0.0–47.7	0.0–91.9		
		☐Thaumatin				
22 EU Member States, Tennant and Bruyninckx, 2017 [68]	Infants, toddlers, other children, adolescents, adults, elderly, very elderly (*n* = NR)	☐Ace-K	-	-	Results are slightly higher than those provided in the ANS Opinion based on the same input data. The incorporation of occurrence results in a significant reduction of intakes, and only the maximum brand loyal scenario for toddlers slightly exceeded the ADI.	Inclusion of occurrence data. A level of 100% occurrence was set for the brand loyal assessment. Intakes were estimated based on summary statistics and MPLs (++). Inability to match FoodEx categories to Regulation (EU) 1333/2008 and Mintel GNDP resulted in inclusion/exclusion of some categories (+/−).
		☐Aspartame	-	-		
		☐Cyclamate	-	-		
		☐Saccharin	-	-		
		☒Steviol	*2.5–75* ^8^	*0–**150*** ^11^		
		☐Sucralose	-	-		
		☐Thaumatin	-	-		

Ace-K = acesulfame-K; ADI = acceptable daily intake; ANS = EFSA Panel on Food Additives and Nutrient Sources added to Food; bw = body weight; CMPA = cow’s milk protein allergy; DNFCS = Dutch National Food Consumption Survey; EFSA = European Food Safety Authority; EU = European Union; FSMP = foods for special medical purposes; GNDP = Global New Product Database; INCA = individual and national food consumption survey; INRAN-SCAI = Italian National Food Consumption Survey; LOR = limit of reporting; MPL = maximum permitted level; *n* = sample size; NCFS = National Children’s Food Survey; ND: not determined; NDNS = National Diet and Nutrition Survey; NPNS = National Pre-School Nutrition Survey; NR = not reported; NS = not specified; NSIFCS = North South Ireland Food Consumption Survey; NTFS = National Teens’ Food Survey; PKU = phenylketonuria; T1D = Type 1 Diabetes; UK = United Kingdom. ^1^ Results are based on ADIs derived by EFSA; figures are **bolded** when the ADI is exceeded; figures are italicized when results as %ADI were calculated based on data reported in the publication (as mg/kg bw/day); ^2^ Average intakes are presented as the mean (actual or adjusted) consumption level; median intakes are presented if mean was not available; adjusted mean value were included in some studies; ^3^ High level consumers are defined as the 95th percentile unless otherwise indicated; ^4^ Comments and uncertainty analysis findings are based on information reported by study authors, or data identified from the study. Sources of under- or over-estimation are identified by (–)/(+); +, ++, +++ are the uncertainties likely to cause small, medium or large overestimates of exposure; −, −− are the uncertainties likely to cause small or medium underestimates of exposure. Information may not be comprehensive for all models available; ^5^ Total population, 99th percentile; ^6^ Intake from food only (exposure from beverages was negligible) evaluated in adults only; ^7^ 97.5th percentile; ^8^ Total population (consumers and non-consumers); ^9^ 90th percentile; ^1^^0^ 99th percentile; ^11^ High level intakes were calculated by adding the highest 95th percentile consumer-only intakes of food categories (number of food categories not specified) to the mean intakes of all other food categories for the total population.

**Table 4:** Estimated Daily Intakes of for Low-/No-Calorie Sweeteners in Latin America.

Country, Reference	Population Group Examined (*n*)	Consumer Daily Intake (%ADI) ^1^			Conclusions ^4^	Comments/Uncertainty Analysis Findings ^5^
		Sweetener Name	Average ^2^	Max ^3^		
Argentina, Cagnasso et al., 2007 [81]	Cohort of children and adolescents attending public and private schools (middle and upper middle class) in Buenos Aires, 3–18 years (*n* = 190)	☒Ace-K	*4.6*	*62.9*	A high proportion of students surveyed (75%) were consumers of non-alcoholic beverages containing sweeteners. The mean estimated intake of all 4 sweeteners was below the ADI. However, 1.5% of the students exceeded the ADI for cyclamate, and 5.2% consumed 50–100% of the ADI from non-alcoholic beverages alone. Given the significant consumption of non-alcoholic beverages, it is recommended that the ADI of non-nutritive sweeteners in children is evaluated and further analyses are conducted to allow results to be extrapolated to the general population.	Sample not nationally representative. Use of actual use level data.Cohort was selected based on the higher risk of exceeding the ADI (+). Intakes were based on the consumption of non-alcohlic beverages only (not total diet) (−). Results were calculated for total population (consumers and non-consumers) (−). Only maximum intakes were examined, not high percentile (+). No account for occurrence (+).
		☒Aspartame	*7*	*52*		
		☒Cyclamate	*23.7*	***172.7***		
		☒Saccharin	*5.6*	*33.4*		
		☐Steviol	-	-		
		☐Sucralose	-	-		
		☐Thaumatin	-	-		
Chile, Durán Agüero et al., 2011 [82]	Cohort of children attending school in the Valparaíso region, 6–14 years (*n* = 281)	☒Ace-K	*11.3*	92.6	All students surveyed were consumers of products containing sweeteners. The mean estimated intake of sweeteners was below the ADI, but for some students, sweetener intake approached the ADI.	Sample not nationally representative. Use of actual use level data. Only maximum intakes were examined, not high percentile (+). No account for occurrence (+).
		☒Aspartame	*11.8*	66		
		☒Cyclamate	*4.5*	74.2		
		☒Saccharin	*0.4*	6		
		☒Steviol	*0*	-		
		☒Sucralose	*18*	82.6		
		☐Thaumatin	-	-		
Chile, Hamilton et al., 2013 [85]	Cohort from the Metropolitan region of Adults, 18–79 years (*n* = 477); children, 6–17 years (*n* = 516); adults with diabetes (Type1/2), 18–79 years (*n* = 155); children with diabetes (Type 1), 3–17 years (*n* = 63)	☒Ace-K	NR ^6^	ND	97.5% of adults and 98.8% of children did not exceed the ADI for any of the sweeteners studied. 5.8% and 25% of diabetic adults and children, repectively, exceeded the ADI for saccharin and cyclamate.	Sample size of diabetic individuals was small. No estimation of high-level intakes (−−). Use of actual use level data. No account for occurrence (+).
		☒Aspartame				
		☒Cyclamate				
		☒Saccharin				
		☒Steviol				
		☒Sucralose				
		☐Thaumatin				
Chile, Durán Agüero et al., 2014 [83]	Cohort of school children from Viña del Mar and Santiago, 10–16 years (*n* = 571)	☒Ace-K	*0.1–0.7*	ND	The majority of students surveyed (96.6%) consumed food products containing sweeteners daily, though the mean estimated intake of the evaluated sweeteners did not exceed the ADI.	Sample not nationally representative. No estimation of high-level intakes (−). Use of actual use level data. No account for occurrence (+).
		☒Aspartame	*3.5–14.0*			
		☒Cyclamate	*0.0–2.3*			
		☒Saccharin	*0.0–42.8*			
		☐Steviol	-			
		☒Sucralose	*7.8–17.5*			
		☐Thaumatin	-			
Chile, Panama, Guatemala, and Peru, Durán Agüero et al., 2015 [86]	Cohort of adults attending university from each country, 18–26 years (*n* = 1224)	☒Ace-K	3.1–7.7	ND	More than 80% of students surveyed consumed products containing the evaluated sweeteners. Mean estimated sweetener intake did not exceed the ADI.	Sample not nationally representative. No estimation of high-level intakes (−). Intakes of cyclamate and saccharin were not derived because both sweeteners are not consumed in Chile, whereas stevia was noted to not be consumed in Panama or Guatamala. Use of actual use level data. No account for occurrence (+).
		☒Aspartame	2.9–4.5			
		☐Cyclamate	-			
		☐Saccharin	-			
		☐Steviol	-			
		☒Sucralose	2.4–9.2			
		☐Thaumatin	-			
Chile, Panama, Guatemala, and Peru, Durán Agüero et al., 2015 [87]	Cohort of adults attending university from each country, 18–26 years (*n* = 1229)	☒Ace-K	*0.0–0.5*	ND	The percentage of consumers of carbonated beverages containing acesulfame-K, aspartame, and sucralose was high (>80%). Mean estimated sweetener intake did not exceed the ADI.	Sample not nationally representative. Intakes were based on the consumption of carbonated beverages only (−). No estimation of high-level intakes (−). Use of actual use level data. No account for occurrence (+).
		☒Aspartame	*0.0–0.6*			
		☐Cyclamate	-			
		☐Saccharin	-			
		☐Steviol	-			
		☒Sucralose	*0.0–0.4*			
		☐Thaumatin	-			
Chile, Durán Agüero et al., 2015 [84]	Cohort of adults attending 4 different universities (first year students, mean age 20.3 to 20.8 years (*n* = 486)	☐Ace-K	-	ND	The percentage of consumers of food and beverages containing stevia was high (69.8%). The mean estimated stevia intake did not exceed the ADI.	Sample not nationally representative. No estimation of high-level intakes (−). Use of actual use level data. No account for occurrence (+).
		☐Aspartame	-			
		☐Cyclamate	-			
		☐Saccharin	-			
		☒Steviol	*6.0–14.0*			
		☐Sucralose	-			
		☐Thaumatin	-			

Ace-K = acesulfame-K; ADI = acceptable daily intake; *n* = sample size; ND: not determined; NR = not reported. ^1^ Results are based on ADIs derived by JECFA, unless otherwise stated; figures are **bolded** when the ADI is exceeded; figures are *italicized* when results for %ADI were calculated based on data reported in the publication (as mg/kg bw/day); ^2^ Average intakes are presented as the mean consumption level; median intakes are presented if mean was not available; ^3^ Maximum intakes (no value reported for high level intakes) in Latin American studies; ^4^ Conclusions related to sweetener intake only are presented; ^5^ Comments and uncertainty analysis findings are based on information reported by study authors, or data identified from the study. Sources of under- or over-estimation are identified by (−)/(+); +, ++, +++ are the uncertainties likely to cause small, medium or large overestimates of exposure; −, −− are the uncertainties likely to cause small or medium underestimates of exposure. Information may not be comprehensive for all models available; ^6^ Results presented on an absolute basis (mg/day) only. Average subject body weight (children, adults) is not reported. However, results as a %ADI were discussed in the paper, as reported in this table.

**Table 5:** Estimated Daily Intakes for Low-/No-Calorie Sweeteners Evaluated or Derived by JECFA.

Country, Reference	Population Group Examined (*n*)	Consumer Daily Intake (%ADI) ^1^			Conclusions	Comments/Uncertainty Analysis Findings ^3^
		Sweetener Name	Average ^2^	High Level		
Global, JECFA, 2009 [92]	Global population (GEMS/Food); Japan (*per capita* disappearance); Japan (*per capita* replacement estimate); US (*per capita* replacement); Diabetic adults; Diabetic child; Non-diabetic child	☐Ace-K	-	-	Replacement estimates were highly conservative, and dietary exposure to steviol glycosides (as steviol) would likely be 20–30% of these values. Actual intakes are likely to be within the ADI range.	Average estimates (GEMS/Food, *per capita* assessments) assumed complete replacement of dietary sugars (++).
		☐Aspartame	-	-		
		☐Cyclamate	-	-		
		☐Saccharin	-	-		
		☒Steviol	*22.5–**145*** ^4^	*37.5–42.5* ^5^		
		☐Sucralose	-	-		
		☐Thaumatin	-	-		
Global, JECFA, 2010 [93]	Australian indivduals, ≥2 years (1995; 2004; 2007), Brazilian individuals (1995), German individuals (1995), Italian teenagers, 13–19 years (1999; 2004), individuals from New Zealand >12 years (2004); Spain, 6–75 years (1996); United Kingdom, aged 1.5–4.5 years (2003)	☐Ace-K	-	-	In some subgroups of populations, primarily children, the ADI of 0–11 mg/kg bw/day was exceeded at high percentiles. A maximum use level of 350 mg/kg also resulted in dietary exposures for high consumers, including children, that were less than the ADI.	Range of different methodologies included in exposure assessments. Some of the consumption data may be out of date (+/−). Older data were based on maximum use levels which were higher than current provisions (+).
		☐Aspartame	-	-		
		☒Cyclamate	*0.5–40.9*	*5.5–**162.7**^6^*		
		☐Saccharin	-	-		
		☐Steviol	-	-		
		☐Sucralose	-	-		
		☐Thaumatin	-	-		

Ace-K = acesulfame-K; ADI = acceptable daily intake; bw = body weight; JECFA = Joint FAO/WHO Expert Committee on Food Additives; US = United States. ^1^ Results are based on ADIs derived by JECFA; figures are **bolded** when the ADI is exceeded; figures are *italicized* when results as %ADI are calculated based on data reported in the publication (as mg/kg bw/day); ^2^ Average intakes are presented as the mean consumption level; median intakes are presented if mean was not available; ^3^ Comments and uncertainty analysis findings are based on information reported by study authors or data identified from the study. Sources of under- or over-estimation are identified by (−)/(+); +, ++, +++ are the uncertainties likely to cause small, medium or large overestimates of exposure; −, −− are the uncertainties likely to cause small or medium underestimates of exposure. Information may not be comprehensive for all models available; ^4^ Intake was determined for the total population (consumers and non-consumers); ^5^ High percentile estimates are provided for diabetic adults, diabetic children and non-diabetic children; ^6^ Includes estimates for the 90th and 95th percentile and maximum intakes.

## Supplementary Materials

The following are available online at http://www.mdpi.com/2072-6643/10/3/357/s1, Table S1: Methodologies Utilized Intake Assessments Conducted Low-/No-Calorie Sweeteners in Asia; Table S2: Methodologies Utilized Intake Assessments Conducted Low-/No-Calorie Sweeteners in Australia/New Zealand; Table S3: Methodologies Utilized Intake Assessments Conducted Low-/No-Calorie Sweeteners in Europe; Table S4: Methodologies Utilized Intake Assessments Conducted Low-/No-Calorie Sweeteners in Latin America; Table S5: Methodologies Utilized Intake Assessments Evaluated or Derived by JECFA.

## Appendix A

Appendix A describes the studies identified in terms of the population group examined and the primary intake assessment inputs for each study. Data are presented in tabular format (Table S1 to S5), organized in ascending order for each region wherein data was identified (Asia, Australia/New Zealand, Europe, Latin America, and global).

In terms of the assessment of inputs, the specific data utilized in each study are summarized, with respect to:

- Food consumption information;
- Chemical concentration data; and
- Exposure assessment method and details.

For the food consumption information, the method of recording consumption data is described. For the chemical concentration data, information is presented on the source of the chemical concentration data (i.e., regulatory limits, reported use levels and/or analytical data), and whether presence data or market share information were included in the model. Finally, the assessment model(s) used in each assessment are described as deterministic, simple distribution and/or probabilistic, with details provided as relevant. The order of the studies in these tables correspond to the tables in the current review and allow an examination of the specific methodologies used in each region as described herein.

## References

[1] World Health Organization (WHO) (2015). Guideline: Sugars Intake for Adults and Children.

[2] World Health Organization (WHO) (2005). Principles of Characterizing and Applying Human Exposure Models.

[3] World Health Organization (WHO) (2009). Dietary of chemicals in food. Principles and Methods for the Risk Assessment of Chemicals in Food.

[4] European Food Safety Authority (EFSA) Food Additives Intake Model (FAIM)—Model 2.0—October 2017. https://www.EFSA.europa.eu/sites/default/files/applications/FAIM-instructions.pdf.

[5] European Food Safety Authority (EFSA) Food Additives Intake Model (FAIM)—Version 1.0—December 2012. https://www.EFSA.europa.eu/sites/default/files/EFSA_rep/blobserver_assets/faimtemplateinstructions.pdf.

[6] European Food Safety Authority (EFSA) Food Additives Intake Model (FAIM)—Version 1.1—July 2013. www.EFSA.europa.eu/sites/default/files/assets/faimtemplate.xls.

[7] European Commission (2001). Report from the Commission on Dietary Food Additive Intake in the European Union.

[8] Arcella D., Soggiu M.E., Leclercq C. (2003). Probabilistic modelling of human exposure to intense sweeteners in Italian teenagers: Validation and sensitivity analysis of a probabilistic model including indicators of market share and brand loyalty. Food Addit. Contam..

[9] Renwick A.G. (2008). The use of a sweetener substitution method to predict dietary exposures for the intense sweetener rebaudioside A. Food Chem. Toxicol..

[10] European Food Safety Authority (EFSA) (2011). Overview of the procedures currently used at EFSA for the assessment of dietary exposure to different chemical substances. EFSA J..

[11] European Food Safety Authority (EFSA) (2006). Guidance of the Scientific Committee on a request from EFSA related to uncertainties in dietary exposure assessment. EFSA J..

[12] Kroes R., Müller D., Lambe J., Löwik M.R.H., van Klaveren J., Kleiner J., Massey R., Mayer S., Urieta I., Verger P. (2002). Assessment of intake from the diet. Food Chem. Toxicol..

[13] Kettler S., Kennedy M., McNamara C., Oberdorfer R., O’Mahony C., Schnabel J., Smith B., Sprong C., Faludi R., Tennant D. (2015). Assessing and reporting uncertainties in dietary exposure analysis: Mapping of uncertainties in a tiered approach. Food Chem. Toxicol..

[14] Scientific Committee on Food (SCF) (2000). Re-Evaluation of Acesulfame K with Reference to the Previous SCF Opinion of 1991 (Expressed on 9 March 2000).

[15] Scientific Committee on Food (SCF) (2000). Revised Opinion on Cyclamic Acid and its Sodium and Calcium Salts (Expressed on 9 March 2000).

[16] JECFA (1991). 3.1.5. Sweetening agents. Acesulfame potassium. Evaluation of Certain Food Additives and Contaminants, Proceedings of the 37th Meeting of the Joint FAO/WHO Expert Committee on Food Additives (JECFA), Geneva, Switzerland, 5–14 June 1990.

[17] JECFA (2010). 3.1.3. Cyclamic acid and its salts: Dietary exposure assessment. Evaluation of Certain Food Additives, Proceedings of the Seventy-First Meeting of the Joint FAO/WHO Expert Committee on Food Additives (JECFA), Geneva, Switzerland, 16–24 June 2009.

[18] JECFA (1986). 3.6. Sweetening agents. Thaumatin. Evaluation of Certain Food Additives and Contaminants, Proceedings of the Twenty-Ninth Meeting of the Joint FAO/WHO Expert Committee on Food Additives (JECFA), Geneva, Switzerland, 3–12 June 1985.

[19] SCF (1989). Report of the Scientific Committee for Food on Sweeteners (opinion expressed on 11 December 1987 and 10 November 1988). Food Science and Techniques.

[20] Liu C.-H., Xie Y.-Y., Sun Y.-M., Zhang M.M., Deng Y.-Y. (2012). Risk assessment for exposure to food additives in preserved fruits among female college students. Xian Dai yu Fang yi Xue Mod. Prev. Med..

[21] Cao P., Ma N., Liang J., Wang X.D., Xu H.B. (2016). Theoretical risk assessment for dietary exposure to sodium cyclamate in Chinese population. Zhongguo Shi Pin Wei Sheng Za Zhi [Chin. J. Food Hyg.].

[22] Singhal P., Mathur P. (2008). Availability and consumption pattern of artificial sweeteners among diabetics, overweight individuals and college girls in Delhi. Indian J. Nutr. Diet..

[23] Sadamasu Y., Mae K., Fujiwara T., Suzuki K., Shindo T., Nakazato M. (2009). Study on daily intake of food additives in citizens of Tokyo (Benzoic acid, sorbic acid, p-hydroxybenzoic acid esters, aspartame, saccharin and acesulfame potassium). Tokyo Toritsu Eisei Kenkyu Nenpo Annu. Rep. Tokyo Metropol. Res. Lab. Public Health.

[24] MHLW (2010). Survey Results of Intake of Food Colours and Preservatives etc., by the Market Bascket Method in 2009.

[25] Kawasaki H., Takagi S., Onishi Y., Urashima Y., Sekini Y., Sato M., Taguchi N., Nishioka C., Yasunaga M., Kawahara R. (2011). Estimation of daily intakes of food additives using the market basket method in Japan (2006–2008). Jpn. J. Food Chem. Saf..

[26] MHLW (2011). Survey Results of Intake of Food Colours and Preservatives etc, by the Market Basket Method in 2010.

[27] MHLW (2012). Survey Results of Intake of Sweeteners by the Market Basket Method in 2011.

[28] Sato K., Takashi K.N., Yoru U. (2013). Estimation of the Intake of Food Additives Based on the Production Volume Survey.

[29] Kumai Y., Hosoki N., Kawashima A., Sekine Y., Hayashi C., Hongo T., Yasunaga M., Ujike A., Nakashim A., Ogata N. (2015). Estimation of daily intakes of food additives in children using market basket method (2014). Jpn. J. Food Chem. Saf..

[30] MHLW (2015). Survey Results of Intake of Preservatives etc, by the Market Basket Method in 2014.

[31] MHLW (2016). Survey Results of Intake of Sweeteners by the Market Basket Method in 2015.

[32] Choi S.H., Lee M.S., Park E.Y., Won J., Kim S.H., Park S.K., Lim H.S. (2011). Assessment of estimated daily intake of sweeteners in the Korean population. Hanguk Sikpum Kwahakhoe Chi [Kor. J. Food Sci. Technol.].

[33] Lee Y.M., Na B.J., Lee Y.S., Kim S.C., Lee D.H., Seo I.W., Choi S.H., Kim D.H., Ha S.D. (2011). Risk assessment of sweeteners in children’s snack. J. Food Hyg. Saf..

[34] Ha M.-S., Ha S.-D., Choi S.-H., Bae D.-H. (2013). Assessment of exposure of Korean consumers to acesulfame K and sucralose using a stepwise approach. Int. J. Food Sci. Nutr..

[35] Ha M.-S., Ha S.-D., Choi S.-H., Bae D.-H. (2013). Assessment of Korean consumer exposure to sodium saccharin, aspartame and stevioside. Food Addit. Contam. Part A Chem. Anal. Control Exp. Risk Assess..

[36] Suh H.-J., Choi S. (2013). Use of sodium saccharin and sucralose in foodstuffs and the estimated daily intakes of both products in Korea. Hanguk Sikpum Kwahakhoe Chi [Kor. J. Food Sci. Technol.].

[37] Kim S.-D., Moon H.K., Lee J.-H., Chang M.-S., Shin Y., Jung S.-O., HYun E.-S., Jo H.-B., Kim J.-H. (2014). Assessment of estimated daily intakes of artificial sweeteners from non-alcoholic beverages in children and adolescents. J. Korean Soc. Food Sci. Nutr..

[38] Suh H.-J., Choi J.C., An D.-J., Choi S., Kim D.Y., Kim A.Y. (2014). Assessment of dietary consumption patterns of aspartame and acesulfame k in the Korean population. J. Korean Soc. Food Sci. Nutr..

[39] Lee Y., Do B., Lee G., Lim H.S., Yun S.S., Kwon H. (2017). Simultaneous determination of sodium saccharin, aspartame, acesulfame-K and sucralose in food consumed in Korea using high-performance liquid chromatography and evaporative light-scattering detection. Food Addit. Contam. Part A Chem. Anal. Control Exp. Risk Assess..

[40] Kim M., Lee G., Lim H.S., Yun S.S., Hwang M., Hong J.H., Kwon H. (2017). Safety assessment of 16 sweeteners for the Korean population using dietary intake monitoring and poundage method. Food Addit. Contam. Part A Chem. Anal. Control Exp. Risk Assess..

[41] National Health and Family Planning Commission of the PRC (2014). Food Safety National Standard Food Additive Standard.

[42] PFA (2006). Prevention of Food Adulteration Act, 1954.

[43] FSANZ (2010). Application A1037-Steviol Glycosides: Increase in Permitted Use Levels: Assessment Report.

[44] FSANZ (2011). Application A1037-Steviol Glycosides: Increase in Permitted Use Levels: Approval Report.

[45] FSANZ (2011). Application A1037-Steviol Glycosides: Increase in Permitted Use Levels: Supporting Document 1.

[46] FSANZ (2015). A1100—Maximum Permitted Level of Acesulphame Potassium in Chewing Gum.

[47] FSANZ (2015). A1100—Maximum Permitted Level of Acesulphame Potassium in Chewing Gum.

[48] FSANZ (2015). A1100—Maximum Permitted Level of Acesulphame Potassium in Chewing Gum.

[49] JECFA (2005). 3.1.6 Steviol glycosides. Evaluation of Certain Food Additives, Proceedings of the Sixty-third Report of the Joint FAO/WHO Expert Committee on Food Additives (JECFA), Geneva, Switzerland, 8–17 June 2004.

[50] JECFA (2006). Steviol glycosides. Safety Evaluation of Certain Food Additives, Proceedings of the Sixty-Third Meeting of the Joint FAO/WHO Expert Committee on Food Additives, Geneva, Switzerland, 8–17 June 2004.

[51] Bemrah N., Leblanc J.C., Volatier J.L. (2008). Assessment of dietary exposure in the French population to 13 selected food colours, preservatives, antioxidants, stabilizers, emulsifiers and sweeteners. Food Addit. Contam. Part B Surveill..

[52] Husøy T., Mangschou B., Fotland T.Ø., Kolset S.O., Notvik Jakobsen H., Tømmerberg I., Bergsten C., Alexander J., Frost Andersen L. (2008). Reducing added sugar intake in Norway by replacing sugar sweetened beverages with beverages containing intense sweeteners-a risk benefit assessment. Food Chem. Toxicol..

[53] Leth T., Jensen U., Fagt S., Andersen R. (2008). Estimated intake of intense sweeteners from non-alcoholic beverages in Denmark, 2005. Food Addit. Contam..

[54] Lino C.M., Costa I.M., Pena A., Ferreira R., Cardoso S.M. (2008). Estimated intake of the sweeteners, acesulfame-K and aspartame, from soft drinks, soft drinks based on mineral waters and nectars for a group of Portuguese teenage students. Food Addit. Contam..

[55] Lino C.M., Pena A. (2010). Occurrence of caffeine, saccharin, benzoic acid and sorbic acid in soft drinks and nectars in Portugal and subsequent exposure assessment. Food Chem..

[56] Mischek D. (2010). Abschätzung der Aufnahme von Süßstoffen über den Konsum von Getränken in Österreich Intake assessment of intense sweeteners from consumption of beverages in Austria. Ernährung.

[57] Hendriksen M.A., Tijhuis M.J., Fransen H.P., Verhagen H., Hoekstra J. (2011). Impact of substituting added sugar in carbonated soft drinks by intense sweeteners in young adults in the Netherlands: Example of a benefit-risk approach. Eur. J. Nutr..

[58] Huvaere K., Vandevijvere S., Hasni M., Vinkx C., Van Loco J. (2012). Dietary intake of artificial sweeteners by the Belgian population. Food Addit. Contam. Part A Chem. Anal. Control Exp. Risk Assess..

[59] Diogo J.S.G., Silva L.S.O., Pena A., Lino C.M. (2013). Risk assessment of additives through soft drinks and nectars consumption on portuguese population: A 2010 survey. Food Chem. Toxicol..

[60] Vin K., Connolly A., McCaffrey T., McKevitt A., O’Mahony C., Prieto M., Tennant D., Hearty A., Volatier J.L. (2013). Estimation of the dietary intake of 13 priority additives in France, Italy, the UK and Ireland as part of the facet project. Food Addit. Contam. Part A Chem. Anal. Control Exp. Risk Assess..

[61] Mancini F.R., Paul D., Gauvreau J., Volatier J.L., Vin K., Hulin M. (2015). Dietary exposure to benzoates (E210–E213), parabens (E214–E219), nitrites (E249–E250), nitrates (E251–E252), BHA (E320), BHT (E321) and aspartame (E951) in children less than 3 years old in France. Food Addit. Contam. Part A Chem. Anal. Control Exp. Risk Assess..

[62] Van Loco J., Vandevijvere S., Cimenci O., Vinkx C., Goscinny S. (2015). Dietary exposure of the Belgian adult population to 70 food additives with numerical ADI. Food Control..

[63] Dewinter L., Casteels K., Corthouts K., Van De Kerckhove K., Van Der Vaerent K., Vanmeerbeeck K., Matthys C. (2016). Dietary intake of non-nutritive sweeteners in type 1 diabetes mellitus children. Food Addit. Contam. Part A Chem. Anal. Control. Exp. Risk Assess.

[64] Martyn D.M., Nugent A.P., McNulty B.A., O’Reilly E., Tlustos C., Walton J., Flynn A., Gibney M.J. (2016). Dietary intake of four artificial sweeteners by Irish pre-school children. Food Addit. Contam. Part A Chem. Anal. Control. Exp. Risk Assess..

[65] O’Sullivan A.J., McKevitt A.I. (2016). Exploring scenarios of dietary exposure to estimate the intake of sucralose by young children with medical conditions. Proc. Nutr. Soc..

[66] Le Donne C., Mistura L., Goscinny S., Janvier S., Cuypers K., D’Addezio L., Sette S., Catasta G., Ferrari M., Piccinelli R. (2017). Assessment of dietary intake of 10 intense sweeteners by the Italian population. Food Chem. Toxicol..

[67] O’Sullivan A.J., Pigat S., O’Mahony C., Gibney M.J., McKevitt A.I. (2017). Longitudinal modelling of the exposure of young uk patients with PKU to acesulfame K and sucralose. Food Addit. Contam. Part A Chem. Anal. Control. Exp. Risk Assess..

[68] Tennant D.R., Bruyninckx C. (2017). The potential application of European market research data in dietary exposure modelling of food additives. Food Addit. Contam. Part A Chem. Anal. Control. Exp. Risk. Assess..

[69] Buffini M., Goscinny S., Van Loco I.J., Nugent A.P., Walton J., Flynn A., Gibney M.J., McNulty B.A. (2017). Dietary intakes of six intense sweeteners by Irish adults. Food Addit. Contam. Part A Chem. Anal. Control Exp. Risk Assess..

[70] EFSA (2013). Scientific Opinion on the re-evaluation of aspartame (E 951) as a food additive. EFSA J..

[71] VKM (2014). Risk Assessments of Cyclamate, Saccharin, Neohesperidine DC, Steviol Glycosides and Neotame from Soft Drinks, “Saft” and Nectar: Opinion of the Panel on Food Additives, Flavourings, Processing Aids, Materials in Contact with Food and Cosmetics; VKM Report 2014: 21.

[72] VKM (2014). Risk Assessments of Aspartame, Acesfulfame K, Sucralose and Benzoic acid from Soft Drinks, “Saft”, Nectar and Flavoured water: Opinion of the Panel on Food Additives, Flavourings, Processing Aids, Materials in Contact with Food and Cosmetics; VKM Report 2014: 26.

[73] EFSA (2015). Scientific opinion on the safety of the extension of use of steviol glycosides (E 960) as a food additive. EFSA J..

[74] EFSA (2015). Scientific Opinion on the safety of the extension of use of thaumatin (E 957). EFSA J..

[75] EFSA (2016). Scientific opinion on the safety of the proposed extension of use of acesulfame K (E 950 in foods for special medical purposes in young children). EFSA J..

[76] EFSA (2016). Safety of the proposed extension of use of sucralose (E 955) in foods for special medical purposes in young children. EFSA J..

[77] EC (2008). Regulation (EC) No 1333/2008 of the European Parliament and of the Council of 16 December 2008 on food additives (L354). Off. J. Eur. Union.

[78] EFSA Food Ingredient Applications: Tools (FAIM). https://www.EFSA.europa.eu/en/applications/foodingredients/tools.

[79] EFSA (2011). The EFSA Comprehensive European Food Consumption Database.

[80] EFSA (2014). Scientific Opinion on the revised exposure assessment of steviol glycosides (E 960) for the proposed uses as a food additive. EFSA J..

[81] Cagnasso C.E., López L.B., Valencia M.E. (2007). Non nutritive sweeteners in non-alcoholic drinks: Estimation of the daily intake in children and adolescents. Arch. Argent. Pediatr..

[82] Durán Agüero S., María Quijada M., Loreto Silva V., Nazarena Almonacid M., María Berlanga Z., María Rodríguez N. (2011). Daily consumption levels of non-nutritive sweeteners in school age children from the Valparaiso region. Rev. Chil. Nutr..

[83] Durán Agüero S., Oñate G., Rivera P.H. (2014). Consumption of non-nutritive sweeteners and nutritional status in 10–16 year old students. Arch. Argent. Pediatr..

[84] Durán Agüero S., Vásquez Leiva A., Morales Illanes G., Schifferli Castro I., Sanhueza Espinoza C., Encina Vega C., Vivanco Cuevas K., Mena Bolvaran F. (2015). Consumo de stevia en estudiantes universitarios chilenos y su asociación con el estado nutricional. Nutr. Hosp..

[85] Hamilton V.V., Guzmán E., Golusda C., Lera L., Cornejo V.E. (2013). Non-caloric sweeteners and daily acceptable intake in adults and children with normal weight and obesity from three different socioeconomic levels, and a diabetic group from Metropolitan region. Rev. Chil. Nutr..

[86] Durán Agüero S., Blanco Batten E., del Pilar Rodríguez Noel M., Cordón Arrivillaga K., Salazar de Ariza J., Record Cornwall J., del Pilar Cereceda Bujaico M., Antezana Alzamora S., Espinoza Bernardo S., Encina Vega C. (2015). Association between non-nutritive sweeteners and obesity risk among university students in Latin America. Rev. Med. Chil..

[87] Durán Agüero S., Cornwall J.R., Encina Vega C., Salazar de Ariza J., Cordón Arrivillaga K., del Pilar Cereceda Bujaico M., Antezana Alzamora S., Espinoza Bernardo S. (2015). Consumption of carbonated beverages with nonnutritive sweeteners in Latin America university students. Nutr. Hosp..

[88] Fakhouri T.H., Kit B.K., Ogden C.L. (2012). Consumption of diet drinks in the United States, 2009–2010. NCHS Data Brief.

[89] Sylvetsky A.C., Welsh J.A., Brown R.J., Vos M.B. (2012). Low-calorie sweetener consumption is increasing in the United States. Am. J. Clin. Nutr..

[90] Piernas C., Ng S.W., Popkin B. (2013). Trends in purchases and intake of foods and beverages containing caloric and low-calorie sweeteners over the last decade in the United States. Pediatr. Obes..

[91] Sylvetsky A.C., Jin Y., Clark E.J., Welsh J.A., Rother K.I., Talegawkar S.A. (2017). Consumption of low-calorie sweeteners among children and adults in the United States. J. Acad. Nutr. Diet..

[92] JECFA (2009). Steviol glycosides. Evaluation of Certain Food Additives, Proceedings of the Sixty-Ninth Report of the Joint FAO/WHO Expert Committee on Food Additives (JECFA), Rome, Italy, 17–26 June 2008.

[93] Codex (2017). Water-based flavoured drinks, including “sport”, “energy”, or “electrolyte” drinks and particulated drinks (14.1.4). Codex General Standard for Food Additives (GSFA) Online Database, Proceedings of the 40th Session of the Codex Alimentarius Commission, Geneva, Switzerland, 17–22 July 2017.

[94] Codex (2017). Codex General Standard for Food Additives (GSFA) Online Database. Proceedings of the 40th Session of the Codex Alimentarius Commission, Geneva, Switzerland, 17–22 July 2017.

[95] WHO (1987). Section 5.5: Setting the ADI. Principles for the Safety Assessment of Food Additives and Contaminants in Food.

[96] SCF (1999). Opinion on Stevioside as a Sweetener (Adopted on 17 June 1999).

[97] Goldsmith L.A. (2000). Acute and subchronic toxicity of sucralose. Food Chem. Toxicol..

[98] Von Rymon Lipinski G., Watson D.H. (2002). Sweeteners (Acesulfame K). Food Chemical Safety. Volume 2: Additives.

[99] MAFF (1990). Intakes of Intense and Bulk Sweeteners in the UK 1987–1988.

[100] Food Standards Agency UK (2001). Diary Survey of the Intake of Intense Sweeteners by Young Children from Soft Drinks.

[101] Renwick A.G., Corti A., Corti B., Karger A.G. (1999). Intake of intense sweeteners. Low-Calorie Sweeteners: Present and Future.

[102] Leth T., Fabricius N., Fagt S. (2007). Estimated intake of intense sweeteners from non-alcoholic beverages in Denmark. Food Addit. Contam..

[103] EC (2004). Directive 2003/115/EC of the European Parliament and of the Council of 22 December 2003 amending Directive 94/35/EC on sweeteners for use in foodstuffs (L24). Off. J. Eur. Union.

[104] EFSA (2017). Statement on approach followed for the refined exposure assessment as part of the safety assessment of food additives under re-evaluation (EFSA Panel on Food Additives and Nutrient Sources Added to Food/ANS) (Question no EFSA-Q-2017-00489, adopted: 30 June 2017). EFSA J..

[105] IBGE (2010). Pesquisa de Orcçamentos Familiares, 2008–2009.

[106] IBGE (2010). Pesquisa de Orçamentos Familiares 2008–2009.

[107] IBGE (2010). Pesquisa De Orçamentos Familiares 2008–2009.

[108] IBGE (2011). Análise do Consumo Alimentar Pessoal no Brasil. Pesquisa de Orçamentos Familiares, 2008–2009.

[109] INSP (2012). Encuesta Nacional de Salud y Nutricion (ENSANUT) 2011–2012.

[110] Fisberg M., Kovalskys I., Gómez G., Rigotti A., Cortés L.Y., Herrera-Cuenca M., Yépez M.C., Pareja R.G., Guajardo V., Zimberg I.Z. (2016). Latin American Study of Nutrition and Health (ELANS): Rationale and study design (ELANS Study Group). BMC Public Health.

[111] HSRC (2012). SANHANES: Health and Nutrition (South African National Health and Nutrition Examination Survey).

[112] Barlow S.M. (2009). Toxicology of food additives. General, Applied and Systems Toxicology.

[113] Reid A.E., Chauhan B.F., Rabbani R., Lys J., Copstein L., Mann A., Abou-Setta A.M., Fiander M., MacKay D.S., McGavock J. (2016). Early exposure to nonnutritive sweeteners and long-term metabolic health: A systematic review. Pediatrics.

[114] Rogers P.J., Hogenkamp P.S., De Graaf C., Higgs S., Lluch A., Ness A.R., Penfold C., Perry R., Putz P., Yeomans M.R. (2016). Does low-energy sweetener consumption affect energy intake and body weight? A systematic review, including meta-analyses, of the evidence from human and animal studies. Int. J. Obes..

[115] Azad M.B., Abou-Setta A.M., Chauhan C., Rabbani R., Lys J., Copstein L., Mann A., Jeyaraman M.M., Reid A.E., Fiande M. (2017). Nonnutritive sweeteners and cardiometabolic health: A systematic review and meta-analysis of randomized controlled trials and prospective cohort studies. CMAJ.

[116] Lohner S., Toews I., Meerpohl J.J. (2017). Health outcomes of non-nutritive sweeteners: Analysis of the research landscape. Nutr. J..

[117] EFSA (2009). General principles for the collection of national food consumption data in the view of a pan-European dietary survey. EFSA J..

