# Supplementary material for: Low-/No-Calorie Sweeteners: A Review of Global Intakes
Source: Nutrients. 2018 Mar 15;10(3):357. doi: 10.3390/nu10030357 (PMC5872775; doi:10.3390/nu10030357)
Supplement: Supplementary file 1 [file nutrients-10-00357-s001.pdf]

Table S1. Methodologies Utilized for Intake Assessments Conducted for Low-/No-Calorie Sweeteners in Asia.

Country Reference	Population Group Examined (*n*)	Sweeteners Investigated	Food Consumption Data	Chemical Data			Assessment Model(s)
				Concentration Data	Presence Data	Market Share Data	Details
China<br>Liu *et al.*, 2012 [20]	Female college students, 18–25 years (*n* = 2044)<br>Cohort of female college students attending 10 different schools in the Guangdong province	☐Ace-K<br>☐Aspartame<br>☒Cyclamate<br>☒Saccharin<br>☐Steviol<br>☐Sucralose<br>☐Thaumatin	Quantitative FFQ	☒MPL<br>☐Reported Use Level<br>☒Analytical Data (*n* = 252)<br><br>Analytical measurement in preserved fruits (*n* = 252) and MPL	☐Yes<br>☒No	☐Yes<br>☒No	☒Deterministic<br>☒Simple Distribution<br>☐Probabilistic<br><br>2 models:<br>(A) Point estimate: average population intake + average sweetener concentration<br>(B) Simple distribution: Individual consumption data + MPL
China<br>Cao *et al.*, 2016 [21]	All ages, ≥2 years; 2 to 3 years; 4–9 years; 10–17 years; 18–59 years; >60 years (*n* = NR)<br>Participants of China Nutrition and Health Survey (2002)	☐Ace-K<br>☐Aspartame<br>☒Cyclamate<br>☐Saccharin<br>☐Steviol<br>☐Sucralose<br>☐Thaumatin	3*24-hour recalls, FFQ (1-year) and/or weighed record included as part of China Nutrition and Health Survey (2002)	☒MPL [1]<br>☐Reported Use Level<br>☐Analytical Data<br><br>GB 2760-2014 - National Health and Family Planning Commission of	☐Yes<br>☒No	☐Yes<br>☒No	☐Deterministic<br>☒Simple Distribution<br>☐Probabilistic<br><br>Individual consumption data + MPL [1]

Country Reference	Population Group Examined (*n*)	Sweeteners Investigated	Food Consumption Data	Chemical Data			Assessment Model(s)
				Concentration Data	Presence Data	Market Share Data	Details
				the PRC, 2014 [40]			
India<br>Singhal and Mathur, 2008 [22]	Cohort of assumed heavy consumers - diabetics (*n* = 72), OW individuals (*n* = 39), and female college students (from 3 colleges; *n* = 47) [2]; Age range NR	☒Ace-K<br>☒Aspartame<br>☐Cyclamate<br>☒Saccharin<br>☐Steviol<br>☒Sucralose<br>☐Thaumatin	Semi-quantitative FFQ	☒MPL (PFA, 2006) [41]<br>☒Reported Use Level (table-top sweeteners only)<br>☐Analytical Data	☐Yes<br>☒No	☐Yes<br>☒No	☐Deterministic<br>☒Simple Distribution<br>☐Probabilistic<br><br>Individual consumption data + MPL/industry use level
Japan<br>Sadamasu *et al.*, 2009 [23]	Participants of Tokyo Metropolitan Health and Nutrition Survey (2004) (age range [3] and sample size NR)	☒Ace-K<br>☒Aspartame<br>☐Cyclamate<br>☒Saccharin<br>☐Steviol<br>☐Sucralose<br>☐Thaumatin	Semi-weighed household dietary record (24 hours)	☐MPL<br>☐Reported Use Level<br>☒Analytical Data	☐Yes<br>☒No	☐Yes<br>☒No	☒Deterministic<br>☐Simple distribution<br>☐Probabilistic<br><br>Average population consumption data + analytical data
Japan<br>MHLW, 2010 [24]	Children, aged 1–6 years (*n* = 2,123) Participants of National Nurtition Survey (2001–2002) and National Health and	☒Ace-K<br>☐Aspartame<br>☐Cyclamate<br>☒Saccharin<br>☐Steviol<br>☐Sucralose<br>☐Thaumatin	Semi-weighed household dietary record (24 hours)	☐MPL<br>☐Reported Use Level<br>☒Analytical Data<br>Average measurement	☐Yes<br>☒No	☐Yes<br>☒No	☒Deterministic<br>☐Simple distribution<br>☐Probabilistic<br><br>Market basket survey: average consumption data + analytical concentration

Country Reference	Population Group Examined (*n*)	Sweeteners Investigated	Food Consumption Data	Chemical Data			Assessment Model(s)
				Concentration Data	Presence Data	Market Share Data	Details
	Nutrition Survey (2003)			Obtained from 6 government- or national-owned research insitutes			
Japan Kawaski *et al.*, 2011 [25]	Adults, ≥20 years (*n* = 28,062) Participants of National Nurtition Survey (2001–2002) and National Health and Nutrition Survey (2003)	☒Ace-K ☒Aspartame ☐Cyclamate ☒Saccharin ☐Steviol ☒Sucralose ☐Thaumatin	Semi-weighed household dietary record (24 hours)	☐MPL ☐Reported Use Level ☒Analytical Data Average measurement obtained from 6 government- or national-owned research insitutes	☐Yes ☒No	☐Yes ☒No	☒Deterministic ☐Simple distribution ☐Probabilistic Market basket survey: average consumption data + mean analytical concentration (with/without positive samples)
Japan MHLW, 2011 [26]	Adults, ≥20 years (*n* = 21,890) Participants of National Health and Nutrition Survey (2004–2006)	☒Ace-K ☐Aspartame ☐Cyclamate ☒Saccharin ☐Steviol ☐Sucralose ☐Thaumatin	Semi-weighed household dietary record (24 hours)	☐MPL ☐Reported Use Level ☒Analytical Data Average measurement obtained from 6	☐Yes ☒No	☐Yes ☒No	☒Deterministic ☐Simple distribution ☐Probabilistic Market basket survey: average consumption data + analytical concentration

Country Reference	Population Group Examined (*n*)	Sweeteners Investigated	Food Consumption Data	Chemical Data			Assessment Model(s)
				Concentration Data	Presence Data	Market Share Data	Details
				government- or national-owned research insitutes			
Japan<br>MHLW, 2012 [27]	Ages 1-6 years; 7-14 years; 15-19 years; ≥20 years; All ages ≥1 years (*n* = 4510)<br>Participants of Special Survey of the Frequency and Intake of Food Consumption (2010)	☒Ace-K<br>☒Aspartame<br>☐Cyclamate<br>☒Saccharin<br>☒Steviol<br>☒Sucralose<br>☐Thaumatin	3-day food diary (repeated 4 times per year)	☐MPL<br>☐Reported Use Level<br>☒Analytical data<br>Average measurement obtained from 6 government- or national-owned research insitutes	☐Yes<br>☒No	☐Yes<br>☒No	☒Deterministic<br>☐Simple distribution<br>☐Probabilistic<br><br>Market basket survey: average consumption data + mean analytical concentration
Japan<br>Sato *et al.*, 2013 [28]	Total population	☒Ace-K<br>☒Aspartame<br>☐Cyclamate<br>☒Saccharin<br>☐Steviol<br>☒Sucralose<br>☐Thaumatin	NA	NA	☐Yes<br>☒No	☐Yes<br>☒No	☒Deterministic<br>☐Simple distribution<br>☐Probabilistic<br><br>Disappearance data: estimated annual shipment volume (2010–2011) × 0.8 (loss during storage/transportation/use)/

**Country Reference**	**Population Group Examined (*n*)**	**Sweeteners Investigated**	**Food Consumption Data**	**Chemical Data**			**Assessment Model(s)**
				**Concentration Data**	**Presence Data**	**Market Share Data**	**Details**
							12,800,000 people (the Japanese population in 2010)/365 days
Japan<br>Kumai *et al.*, 2015 [29]; MHLW, 2015 [30]	Children 1-6 years (*n* = 227)<br>Participants of Special Survey of the Frequency and Intake of Food Consumption (2010)	☒Ace-K<br>☐Aspartame<br>☐Cyclamate<br>☐Saccharin<br>☐Steviol<br>☒Sucralose<br>☐Thaumatin	3-day food diary (repeated 4 times per year)	☐MPL<br>☐Reported Use Level<br>☒Analytical data<br>Average measurement obtained from 6 government- or national-owned research insitutes	☐Yes<br>☒No	☐Yes<br>☒No	☒Deterministic<br>☐Simple distribution<br>☐Probabilistic<br><br>Market basket survey: average consumption data + mean analytical concentration
Japan<br>MHLW, 2016 [31]	Ages 1-6 years; 7-14 years; 15-19 years; ≥20 years; All ages ≥1 years (*n* = 4,510)<br>Participants of Special Survey of the Frequency and Intake of Food Consumption (2010)	☒Ace-K<br>☒Aspartame<br>☐Cyclamate<br>☒Saccharin<br>☒Steviol<br>☒Sucralose<br>☐Thaumatin	3-day food diary (repeated 4 times per year)	☐MPL<br>☐Reported Use Level<br>☒Analytical data<br>Average measurement obtained from 6 government- or national-owned	☐Yes<br>☒No	☐Yes<br>☒No	☒Deterministic<br>☐Simple distribution<br>☐Probabilistic<br><br>Market basket survey: average consumption data + mean analytical concentration

Country Reference	Population Group Examined (*n*)	Sweeteners Investigated	Food Consumption Data	Chemical Data: Concentration Data	Chemical Data: Presence Data	Chemical Data: Market Share Data	Assessment Model(s) Details
				research insitutes			
Korea<br>Choi *et al.*, 2011 [32]	All ages (age range and sample size NR)<br>Participants of KNHANES (2005)	☒Ace-K<br>☒Aspartame<br>☐Cyclamate<br>☒Saccharin<br>☐Steviol<br>☒Sucralose<br>☐Thaumatin	24-hour recall	☐MPL<br>☐Reported Use Level<br>☒Analytical data (*n* = 584)<br><br>Collected from department stores, spermarkets, and convenience stores	☐Yes<br>☒No	☐Yes<br>☒No	☒Deterministic<br>☐Simple Distribution<br>☐Probabilistic<br><br>Food consumption + mean sweetener concentration
Korea<br>Lee *et al.*, 2011 [33]	Children and adolescents (*n* = 6625); 0–6 years; 7–12 years; 13–19 years<br>Participants of a national dietary survey, Dietary Intake Survey of Infant, Children	☒Ace-K<br>☒Aspartame<br>☐Cyclamate<br>☒Saccharin<br>☐Steviol<br>☒Sucralose<br>☐Thaumatin	2× 24-hour recall	☐MPL<br>☐Reported Use Level<br>☒Analytical data (*n* = 92)<br><br>Snacks targeted at children	☐Yes<br>☒No	☐Yes<br>☒No	☐Deterministic<br>☒Simple Distribution<br>☐Probabilistic<br><br>Individual consumption data + mean sweetener concentration

Country Reference	Population Group Examined (*n*)	Sweeteners Investigated	Food Consumption Data	Chemical Data			Assessment Model(s)
				Concentration Data	Presence Data	Market Share Data	Details
	and Adolescents (2007–2009)						
Korea<br>Ha *et al.*, 2013a<br>[34]	Total population	☒Ace-K<br>☐Aspartame<br>☐Cyclamate<br>☐Saccharin<br>☐Steviol	Budget method	☒MPL[4]<br>☐Reported Use Level<br>☐Analytical data	☐Yes<br>☒No	☐Yes<br>☒No	☒Deterministic<br>☐Simple Distribution<br>☐Probabilistic<br><br>Budget method
	All ages, 1 to >65 years (*n* = 8081)<br>Participants of KNHANES who consumed intense sweeteners during the 24-hour recall (2009)	☒Sucralose<br>☐Thaumatin	24-hour recall	☐MPL<br>☐Reported Use Level<br>☒Analytical data Analytical data (*n* = 605)<br><br>Collected from Korean grocery stores and markets	☐Yes<br>☒No	☐Yes<br>☒No	☐Deterministic<br>☒Simple Distribution<br>☐Probabilistic<br><br>2 scenarios:<br>(A) Individual consumption data + mean sweetener concentration (all samples)<br>(B) Individual consumption data + mean sweetener concentration for positive samples
Korea<br>Ha *et al.*, 2013b<br>[35]	All ages, 1 to >65 years (*n* = 8,081)<br>Participants of KNHANES who consumed intense sweeteners during	☐Ace-K<br>☒Aspartame<br>☐Cyclamate<br>☒Saccharin<br>☒Stevioside<br>☐Sucralose<br>☐Thaumatin	24-hour recall	☐MPL<br>☐Reported Use Level<br>☒Analytical data (*n* = 541)	☐Yes<br>☒No	☐Yes<br>☒No	☐Deterministic<br>☒Simple Distribution<br>☐Probabilistic<br><br>2 scenarios:

Country Reference	Population Group Examined (*n*)	Sweeteners Investigated	Food Consumption Data	Chemical Data			Assessment Model(s)
				Concentration Data	Presence Data	Market Share Data	Details
	the 24-hour recall (2009)			Collected from Korean grocery stores and markets based on permitted use of sweeteners and consumption in KNHANES 2009			(A) Individual consumption data + mean sweetener concentration (all samples) (B) Individual consumption data + mean sweetener concentration for positive samples
Korea Suh and Choi, 2013 [36]	All ages (sample size NR); 1–2 years; 3–6 years; 7–12 years; 13–19 years; 20–29 years; 30–39 years; 40–49 years; 50–64 years; >65 years Participants of KNHANES (2010)	☐Ace-K ☐Aspartame ☐Cyclamate ☒Saccharin ☐Steviol ☒Sucralose ☐Thaumatin	24-hour recall	☐MPL ☐Reported Use Level ☒Analytical data (n=339 for saccharin and 590 for sucralose) Domestic and imported products	☐Yes ☒No	☐Yes ☒No	☒Deterministic ☐Simple Distribution ☐Probabilistic Summary statistics + mean sweetener concentration
Korea Kim *et al.*, 2014 [37]	Children and adolescents, aged 1-19 years (*n* = 6082)	☒Ace-K ☒Aspartame ☐Cyclamate ☐Saccharin	24-hour recall	☐MPL ☐Reported Use Level	☐Yes ☒No	☐Yes ☒No	☐Deterministic ☒Simple Distribution ☒Probabilistic

Country Reference	Population Group Examined (*n*)	Sweeteners Investigated	Food Consumption Data	Chemical Data			Assessment Model(s)
				Concentration Data	Presence Data	Market Share Data	Details
	Participants of KNHANES (2007–2009)	☐Steviol<br>☒Sucralose<br>☐Thaumatin		☒Analytical data (*n* = 738)<br><br>Non-alcoholic beverages only			2 scenarios:<br>(A) Individual consumption data + mean sweetener concentration<br>(B) Individual consumption data + range of sweetener concentrations
Korea<br>Suh *et al.*, 2014<br>[38]	All ages (sample size NR); 1–2 years; 3–6 years; 7–12 years; 13–19 years; 20–29 years; 30–39 years; 40–49 years; 50–64 years; >65 years<br>Participants of KNHANES (2010)	☒Ace-K<br>☒Aspartame<br>☐Cyclamate<br>☐Saccharin<br>☐Steviol<br>☐Sucralose<br>☐Thaumatin	24-hour recall	☐MPL<br>☐Reported Use Level<br>☒Analytical data (*n* = 247 for aspartame and 305 for ace-K)<br><br>Domestic and imported products	☐Yes<br>☒No	☐Yes<br>☒No	☒Deterministic<br>☐Simple Distribution<br>☐Probabilistic<br><br>Summary statistics + mean sweetener concentration
Korea<br>Lee *et al.*, 2017<br>[39]	All ages (*n* = 34,706)<br>Participants of KNHANES (2010–2014)	☒Ace-K<br>☒Aspartame<br>☐Cyclamate<br>☒Saccharin<br>☐Steviol<br>☒Sucralose<br>☐Thaumatin	24-hour recall	☐MPL<br>☐Reported Use Level<br>☒Analytical data (*n* = >900)<br><br>Domestic and	☐Yes<br>☒No	☐Yes<br>☒No	☐Deterministic<br>☒Simple Distribution<br>☐Probabilistic<br><br>Individual consumption data + Mean sweetener concentration of positive samples

Country Reference	Population Group Examined (*n*)	Sweeteners Investigated	Food Consumption Data	Chemical Data: Concentration Data	Chemical Data: Presence Data	Chemical Data: Market Share Data	Assessment Model(s) Details
				imported products			
Korea<br><br>Kim *et al.*, 2017 [42]	All ages; <2 years; 3–6 years; 7–12 years; 13–19 years; 20–64 years; >65 years (*n* = 20,788)<br><br>Participants of KNHANES (2010–2013)	☒Ace-K<br>☒Aspartame<br>☐Cyclamate<br>☒Saccharin<br>☐Steviol<br>☒Sucralose<br>☐Thaumatin	24-hour recall	☐ MPL<br>☐Reported Use Level<br>☒ Analytical data (*n* = >900)<br><br>Lee *et al.*, 2017 [39]	☐Yes<br>☒No	☐Yes<br>☒No	☐Deterministic<br>☒Simple Distribution<br>☐Probabilistic<br><br>Individual consumption data + mean sweetener concentration of positive samples
	Total population	☐Ace-K<br>☐Aspartame<br>☐Cyclamate<br>☐Saccharin<br>☒Steviol<br>☐Sucralose<br>☒Thaumatin	NA	NA	☐Yes<br>☒No	☐Yes<br>☒No	☒Deterministic<br>☐Simple Distribution<br>☐Probabilistic<br><br>Poundage data: Total production, import, and export of food products and food additives

Ace-K = acesulfame-K; FFQ = food frequency questionnaire; KNHANES = Korea National Health and Nutrition Examination Survey; MPL = maximum permitted level; *n* = sample size; NA = not Applicable; NR = not reported; OW = overweight; PRC = People's Republic of China. [1] Value reported as maximum residue limit (MRL) in the publication; however, the MPL is the standard terminology for food additives, and used herein. [2] Number of individuals (n) listed includes only 'regular consumers' of artificial sweeteners (defined as individuals consuming artificial sweeteners ≥1 time a week); intakes by 'occasional consumers' (defined as individuals consuming once a fortnight or less) were not examined. [3] Study does not specifically state the age group investigated; however intakes were expressed for a 50 kg individual, which aligns with the default value for adults utilized for other Japanese studies. [4] The second highest MPL was selected for solid foods as highest MPL was established for 'sugar'.

Table S2. Methodologies Utilized for Intake Assessments Conducted for Low-/No-Calorie Sweeteners in Australia/New Zealand.

Country<br><br>Reference	Population Group Examined (*n*)	Sweeteners Investigated	Food Consumption Data	Chemical Data<br><br>Concentration Data[1]	 <br>Presence Data	 <br>Market Share Data	Assessment Model(s)<br><br>Details
Australia and New Zealand<br><br>FSANZ, 2010, 2011a,b [43–45]	Australian children, 2–16 years (*n* = 4487) Participants of ANNPAS (2007)<br><br>General Australian population, ≥17 years (*n* = NR) Participants of ANNS (1995)<br><br>New Zealand children, 5–14 years (*n* = 3275) Participiptans of NZNNS (2002)<br><br>General New Zealand population, ≥15 years (*n* = 4636)	☐Ace-K<br>☐Aspartame<br>☐Cyclamate<br>☐Saccharin<br>☒Steviol<br>☐Sucralose<br>☐Thaumatin	24-hour recall	☒MPL + proposed amendments [2]<br>☐Reported Use Level<br>☐Analytical data	☐Yes<br>☒No	☒Yes<br>☐No<br><br>Assumed 30% market uptake (30% of the MPL)	☐Deterministic<br>☒Simple Distribution<br>☐Probabilistic<br><br>3 scenarios:<br>(A) Individual consumption data + MPL + 30% market share for all food categories<br>(B) Individual consumption data + MPL + 30% market share for all food categories except for water based flavoured drinks<br>(C) Individual consumption data + MPL + 30% market share for all food categories except for flavoured milk products (including yoghurt)

**Country**<br>**Reference**	**Population Group Examined (*n*)**	**Sweeteners Investigated**	**Food Consumption Data**	**Chemical Data**			**Assessment Model(s)**
				**Concentration Data**[1]	**Presence Data**	**Market Share Data**	**Details**
Australia and New Zealand<br><br>FSANZ, 2015a,b,c [46–48]	Participants of NZNNS (1997)<br>Australian children, 2–6 years (*n* = 779) and 7–11 years (*n* = 802); general Australian population, ≥2 years (*n* = 12,153) and ≥12 years (*n* = 10,572)<br>Participants of ANNPAS (2011–2012)<br><br>New Zealand children, 5–14 years (*n* = 3275)<br>Participants of NZNNS (2002)	☒Ace-K<br>☐Aspartame<br>☐Cyclamate<br>☐Saccharin<br>☐Steviol<br>☐Sucralose<br>☐Thaumatin	24-hour recall	☒MPL + proposed amendments [3]<br>☐Reported Use Level<br>☐Analytical data	☐Yes<br>☒No	☐Yes<br>☒No	☐Deterministic<br>☒Simple Distribution<br>☐Probabilistic<br><br>2 scenarios:<br>(A) Individual consumption data + MPL for chewing gum only<br>(B) Combined estimated dietary exposure from chewing gum and 2004 sweetener survey

**Country** **Reference**	**Population Group Examined (*n*)**	**Sweeteners Investigated**	**Food Consumption Data**	**Chemical Data**			**Assessment Model(s)** **Details**
				**Concentration Data**[1]	**Presence Data**	**Market Share Data**	
	General New Zealand population, ≥15 years (*n* = 4721) Participants of NZANS (2008–2009)						

Ace-k = acesulfame-k; ANNS = Australian National Nutrition Survey; ANNPAS = Australian National Nutrition and Physical Activity Survey; MPL = maximum permitted level; *n* = sample size; NZANS = New Zealand Adult Nutrition Survey; NZNNS = New Zealand National Children's Nutrition Survey. [1] Existing MPLs are specified in Schedule 15 of the Food Standards Code. [2] Amendments proposed by applicant: ice cream and edible ices 64 mg/kg; water based flavored drinks 160 mg/kg; formulated beverages 160 mg/kg, soy beverage, unflavored 65 mg/kg; soy beverage, flavored 175 mg/kg. [3] Amendments proposed by applicant: intensely sweetened chewing gum 5000 mg/kg.

Table S3. Methodologies Utilized for Intake Assessments Conducted for Low-/No-Calorie Sweeteners in Europe.

Country Reference	Population Group Examined (*n*)	Sweeteners Investigated	Food Consumption Data	Chemical Data			Assessment Model(s)
				Concentration Data [1]	Presence Data	Market Share Data	Details
Portugal<br>Lino *et al.*, 2008<br>[54]	Adolescents, 13–15 years (*n* = 65). Cohort of students attending a public high school in Coimbra (2006–2007) and total population	☒Ace-K<br>☒Aspartame<br>☐Cyclamate<br>☐Saccharin<br>☐Steviol<br>☐Sucralose<br>☐Thaumatin	Annual intake of soft drinks and nectars (2006) per Portuguese inhabitant	☐MPL<br>☐Reported Use Level<br>☒Analytical Data (*n* = 48, samples collected in school canteen and supermarkets in 2006–2007)	☐Yes<br>☒No	☐Yes<br>☒No	☒Deterministic<br>☐Simple Distribution<br>☐Probabilistic<br><br>Daily intake + Mean concentration / Default body weight
Denmark<br>Leth *et al.*, 2008<br>[53]	Total population 1–80 years (*n* = 3,098); young children 1–3 years (*n* = 278); children 4–6 years (*n* = 366); children 7–10 years (*n* = 376)	☒Ace-K<br>☒Aspartame<br>☒Cyclamate<br>☒Saccharin<br>☐Steviol<br>☐Sucralose<br>☐Thaumatin	Information on consumption of non-alcoholic beverages collected via 7-day food diary	☐MPL<br>☐Reported Use Level<br>☒Analytical Data (*n* = 177, collected by the Danish Veterinary and Food Administration, 2005-2006)	☐Yes<br>☒No	☐Yes<br>☒No	☐Deterministic<br>☒Simple Distribution<br>☐Probabilistic<br><br>2 scenarios:<br>(A) Individual consumption data + mean concentration of positive samples<br>(B) Individual consumption data + mean concentration of all samples

Country Reference	Population Group Examined (*n*)	Sweeteners Investigated	Food Consumption Data	Chemical Data			Assessment Model(s)
				Concentration Data [1]	Presence Data	Market Share Data	Details
Norway<br>Husøy *et al.*, 2008 [52]	Participants of Danish Dietary Survey (1995) Participants from 4 national dietary surveys Young children, aged 1 year and 2 years from Spedkost (*n* = 1204) and Småbarnskost (*n* = 1720), repsectively; Children, 4-, 9-, and 13 years (*n* = 2215) from Ungkost; Adults, 16–79 years (*n* = 2672) from Norkost food survey (1997); and Adults, aged 16–80 years (*n* =	☒Ace-K<br>☒Aspartame<br>☒Cyclamate<br>☒Saccharin<br>☐Steviol<br>☐Sucralose<br>☐Thaumatin	Information on consumption of beverages and foods [2] from: Semi-quantitative FFQ (1–2 year olds); 4-day food diary (4-,9-, 13-year olds); FFQ (16–79 years); FFQ (16–80 years)	☐MPL<br>☒Reported Use Level<br>☒Analytical Data<br><br>Industry use levels collected by the Norwegian food safety authority (2004)(beverages); Analytical data collected by Bergsten, 1998 (food products)	☐Yes<br>☒No	☒Yes<br>☐No<br><br>For beverages, chemical concentration weighted according to content and sales volumes provided by food manufacturers	☐Deterministic<br>☒Simple Distribution<br>☐Probabilistic<br><br>Individual consumption data + average weighted concentration

Country Reference	Population Group Examined (*n*)	Sweeteners Investigated	Food Consumption Data	Chemical Data			Assessment Model(s)
				Concentration Data [1]	Presence Data	Market Share Data	Details
	1375) from Omnibus survey (1997)						
France Bemrah *et al.*, 2008 [51]	Participants of national dietary survey (INCA1, 1998-1999) All ages (*n* = 3033); children and young teenagers, 3–14 years (1018); adults , ≥15 years (*n* = 1985)	☐Ace-K ☐Aspartame ☒Cyclamate ☐Saccharin ☐Steviol ☐Sucralose ☐Thaumatin	7-day food diary	☐MPL ☒Reported Use Level ☐Analytical Data<br>Industry use levels collected by the DGCCRF (2002–2005)	☐Yes ☒No	☐Yes ☒No	☐Deterministic ☒Simple Distribution ☐Probabilistic<br>2 scenarios: (1) Individual consumption data + Mean concentration of all samples (2) Individual consumption data + Mean concentration of positive samples
Austria Mischek, 2010 [56]	National consumption data available from Australian Nutrition Report 2003 [3] for Preschool children (3–6 years) and adults (19–65) (*n*	☒Ace-K ☒Aspartame ☒Cyclamate ☒Saccharin ☐Steviol ☐Sucralose ☐Thaumatin	3-day weighted record (preschool childern), 24-hour recall (adults)	☐MPL ☐Reported Use Level ☒Analytical Data (*n* = 159, beverage samples collected between 2006–2008)	☐Yes ☒No	☐Yes ☒No	☒Deterministic ☐Simple Distribution ☐Probabilistic<br>Consumption data for population group + average concentration data/default body weight

Country Reference	Population Group Examined (*n*)	Sweeteners Investigated	Food Consumption Data	Chemical Data			Assessment Model(s)
				Concentration Data [1]	Presence Data	Market Share Data	Details
	= 151 and 2581, respectively)						
Portugal<br>Lino and Pena, 2010 [51]	Total population	☐Ace-K<br>☐Aspartame<br>☐Cyclamate<br>☒Saccharin<br>☐Steviol<br>☐Sucralose<br>☐Thaumatin	Annual intake of traditional soft drinks and soft drinks based on mineral water and nectars per Portuguese inhabitant (2006)	☐MPL<br>☐Reported Use Level<br>☒Analytical Data (*n* = 48)<br><br>Purchased in the central zone of Portugal (2006–2007)	☐Yes<br>☒No	☐Yes<br>☒No	☒Deterministic<br>☐Simple Distribution<br>☐Probabilistic<br><br>Daily intake + Mean concentration / Default body weight
Netherlands<br>Hendriksen *et al.*, 2011 [57]	Young adults, 19–30 years (*n* = 750)<br>Participants of DNFCS 2007–2010	☒Ace-K<br>☒Aspartame<br>☒Cyclamate<br>☒Saccharin<br>☐Steviol<br>☐Sucralose<br>☐Thaumatin	Data on carbonated soft drinks from 2 24-h dietary recalls	☐MPL<br>☐Reported Use Level<br>☒Analytical Data<br><br>Collected by the Dutch Food Safety Auhtority (2006 and 2008)	☐Yes<br>☒No	☐Yes<br>☒No	☐Deterministic<br>☒Simple Distribution<br>☐Probabilistic<br><br>Individual consumption data + Actual concentrations (or average, if not available)
Belgium<br>Huvaere *et al.*, 2012 [58]	Adults, aged ≥15 years (*n* = 3,083), including	☒Ace-K<br>☒Aspartame<br>☒Cyclamate<br>☒Saccharin	2× 24-hour recall	☒MPL<br>☐Reported Use Level	☐Yes<br>☒No	☐Yes<br>☒No	☐Deterministic<br>☒Simple Distribution<br>☐Probabilistic

Country Reference	Population Group Examined (*n*)	Sweeteners Investigated	Food Consumption Data	Chemical Data			Assessment Model(s)
				Concentration Data [1]	Presence Data	Market Share Data	Details
	diabetics (*n* = 428) Participants of the Belgian Food Consumption Survey (dating from 2004)	☐Steviol ☒Sucralose ☐Thaumatin		☒Analytical Data (*n* = 119) Sweetened products collected from major supermarkets in Belgium (2009–2010)			2 scenarios: (A) Individual consumption data + MPL (B) Individual consumption data + Mean concentration of positive samples
Portugal Diogo *et al.*, 2013 [59]	Total population	☒Ace-K ☒Aspartame ☐Cyclamate ☒Saccharin ☐Steviol ☐Sucralose ☐Thaumatin	Annual intake of traditional soft drinks and soft drinks based on mineral water and nectars per Portuguese inhabitant (2010)	☐MPL ☐Reported Use Level ☒Analytical Data (*n* = 78) Purchased in central zone of Portugal (2010)	☐Yes ☒No	☐Yes ☒No	☒Deterministic ☐Simple Distribution ☐Probabilistic Daily intake + Mean concentration / Default body weight
France, Italy, UK, Ireland Vin *et al.*, 2013 [60]	France - Participants of the INCA 2 (2005-2007) aged 3-79 years (*n* = 4079)	☒Ace-K ☒Aspartame ☐Cyclamate ☐Saccharin ☐Steviol ☐Sucralose	7-day diary (French survey); 3-day diary (Italian survey); 4-day or 7-day diaries (UK	☒MPL ☒Reported Use Level (collected by Food Drink Europe) ☐Analytical Data	☐Yes ☒No	☐Yes ☒No	☐Deterministic ☒Simple Distribution ☒Probabilistic 2 scenarios:

Country Reference	Population Group Examined (*n*)	Sweeteners Investigated	Food Consumption Data	Chemical Data			Assessment Model(s)
				Concentration Data [1]	Presence Data	Market Share Data	Details
	Italy - Participants of the INRAN-SCAI (2005–2006), aged 1 mth to 97 years (*n* = 3323)<br>UK - Participants of the UK NDNS (1992–2001), aged 1.5 to >65 years (*n* = 6787)<br>Ireland, Participants of the NSIFCS (1997–1999), NCFS (2003–2004), or NTFS (2005–2006), aged 5–64 years (*n* = 2414)	☐Thaumatin	survey); 7-day diary (Irish surveys)				(A) Individual consumption data + MPL<br>(B) Individual consumption data + range of sweetener concentrations
Norway<br>VKM, 2014a [71]	Children, 2 years (*n* = 1674)	☐Ace-K<br>☐Aspartame<br>☒Cyclamate	Semi-quantitative	☒MPL<br>☒Reported Use Level (2012)	☒Yes<br>☐No	☒Yes<br>☐No	☐Deterministic<br>☒ Simple Distribution<br>☐ Probabilistic

Country Reference	Population Group Examined (*n*)	Sweeteners Investigated	Food Consumption Data	Chemical Data			Assessment Model(s)
				Concentration Data [1]	Presence Data	Market Share Data	Details
	Participants of Småbarnskost (2006–2007) Young women, 18–29 years (*n* = 143); Young men, 18–29 years (*n* = 138); 30–70 years (*n* = 782); Men 30–70 years (*n* = 724) Participants of Norkost 3 (2010–2011)	☒Saccharin<br>☒Steviol<br>☐Sucralose<br>☐Thaumatin	FFQ; 2× 24-hour recall	☐Analytical Data<br><br>Soft drinks, "saft", nectar, and flavoured water	Collected from industry (2012)	Annual sales volumes (L/year) (2012)	6 scenarios:<br>(A) Individual consumption data + mean concentration + occurence data + market share data<br>(B) Individual consumption data + mean concentration + market share data<br>(C–cyclamate and saccharin) Individual consumption data + highest reported concentration + occurence data<br>(D–cyclamate and saccharin) Individual consumption data + highest reported concentration<br>(E–steviol glycosides) Individual consumption data + MPL + occurence data<br>(D–steviol glycosides) Individual consumption data + MPL
Norway<br>VKM, 2014b [72]	Children, 2 years (*n* = 1674)	☒Ace-K<br>☒Aspartame<br>☐Cyclamate	Semi-quantitative	☐MPL<br>☒Reported Use Level (2012)	☒Yes<br>☐No	☒Yes<br>☐No	☐Deterministic<br>☒ Simple Distribution<br>☐ Probabilistic

Country Reference	Population Group Examined (*n*)	Sweeteners Investigated	Food Consumption Data	Chemical Data			Assessment Model(s)
				Concentration Data [1]	Presence Data	Market Share Data	Details
	Participants of Småbarnskost (2007) Young women, 18–29 years (*n* = 143); Young en, 18–29 years (*n* = 138); 30–70 years (*n* = 782); Men 30-70 years (*n* = 724) Participants of Norkost 3 (2010–2011)	☐Saccharin<br>☐Steviols<br>☒Sucralose<br>☐Thaumatin	FFQ; 2× 24-hour recall	☐Analytical Data<br><br>Soft drinks, "saft", nectar, and flavoured water	Collected from industry (2012)	Annual sales volumes (L/year) in 2012	4 scenarios:<br>(A) Individual consumption data + mean concentration + occurence data + market share data<br>(B) Individual consumption data + mean concentration + market share data<br>(C) Individual consumption data + highest reported concentration + occurence data<br>(D) individual consumption data + highest reported concentration
France<br><br>Mancini *et al.*, 2015 [61]	Children,1–4 months (*n* = 124); 5–6 months (127); 7–12 months (*n* = 195); 13–36 months (*n* = 259) Participants of the BEBE-SFAE	☐Ace-K<br>☒Aspartame<br>☐Cyclamate<br>☐Saccharin<br>☐Steviol<br>☐Sucralose<br>☐Thaumatin	3-day diary	☒MPL<br>☐Reported Use Level<br>☐Analytical Data	☐Yes<br>☒No	☐Yes<br>☒No	☐Deterministic<br>☒Simple Distribution<br>☐Probabilistic<br><br>Individual consumption data + MPL

Country Reference	Population Group Examined (*n*)	Sweeteners Investigated	Food Consumption Data	Chemical Data			Assessment Model(s)
				Concentration Data [1]	Presence Data	Market Share Data	Details
	dietary survey (2005)						
Belgium<br><br>Van Loco *et al.*, 2015 [62]	Toddlers, children, adolescents, adults, the elderly (FAIM V1.1) (*n* = NR)	☒Ace-K<br>☒Aspartame<br>☒Cyclamate<br>☒Saccharin<br>☐Steviols<br>☒Sucralose<br>☐Thaumatin	FAIM-V1.1 tool	☒MPL<br>☐Reported Use Level<br>☐Analytical Data	☐Yes<br>☒No	☐Yes<br>☒No	☒Deterministic<br>☐Simple Distribution<br>☐Probabilistic<br><br>Summary statistics + MPL
Belgium<br><br>Dewinter *et al.*, 2016 [63]	Children and adolescents with T1D (*n* = 103); 4–6 years (*n* = 9); 7–12 years (*n* = 35); 13–18 years (*n* = 59) Cohort of T1D patients from the Pediatrics Department of the University Hospitals Leuven (2014)	☒Ace-K<br>☒Aspartame<br>☒Cyclamate<br>☒Saccharin<br>☒Steviol<br>☒Sucralose<br>☐Thaumatin	FFQ	☒MPL<br>☐Reported Use Level<br>☒Analytical Data (collected by Huvaere *et al.*, 2012) [58]	☐Yes<br>☒No	☐Yes<br>☒No	☐Deterministic<br>☒Simple Distribution<br>☐Probabilistic<br><br>2 scenarios:<br>(A) Individual consumption data + MPL<br>(B) Individual consumption data + Mean concentration of positive samples or MPL (stevia only)

Country Reference	Population Group Examined (*n*)	Sweeteners Investigated	Food Consumption Data	Chemical Data			Assessment Model(s)
				Concentration Data [1]	Presence Data	Market Share Data	Details
Ireland<br><br>Martyn *et al.*, 2016 [64]	Toddlers and Children ages 1–4 years (*n* = 500) Participants of the NPNS(2011–2012)	☒Ace-K<br>☒Aspartame<br>☐Cyclamate<br>☒Saccharin<br>☐Steviol<br>☒Sucralose<br>☐Thaumatin	4-day diary	☒MPL<br>☐Reported Use Level<br>☒Analytical Data<br><br>FSAI chemical Sampling Program (2008-2011)	☒Yes<br>☐No<br><br>Irish National Food Ingredient Database V4	☐Yes<br>☒No	☐Deterministic<br>☒Simple Distribution<br>☒Probabilistic<br><br>4 scenarios:<br>(A) Individual consumption data + MPL<br>(B) Individual consumption data + MPL + occurence<br>(C) Individual consumption data + range of concentrations<br>(D) Individual consumption data + range of concentrations + occurence
Ireland<br><br>O’Sullivan *et al.*, 2016 [65]	Particpants of the NPNS (2010–2011), aged 1–3 years (*n* = 376), used as a surrogate for children with PKU and CMPA	☒Ace-K<br>☒Aspartame<br>☐Cyclamate<br>☐Saccharin<br>☐Steviol<br>☒Sucralose<br>☐Thaumatin	4-day diary	☒MPL<br>☒Reported Use Level (FSMPs, provided by Nutricia)<br>☐Analytical Data	☒Yes<br>☐No<br><br>Health Service Exceutive (HSE) list of Reimbursable Items	☐Yes<br>☒No	☐Deterministic<br>☒Simple Distribution<br>☒Probabilistic<br><br>5 scenarios[4]:<br>(1) Individual consumption data + MPL + 75% adherence<br>(2a) Individual consumption data+ range of concentrations[5] + 75% adherence

Country Reference	Population Group Examined (*n*)	Sweeteners Investigated	Food Consumption Data	Chemical Data			Assessment Model(s)
				Concentration Data [1]	Presence Data	Market Share Data	Details
							(2b) Individual consumption data+ range of concentrations[5] + 50% adherence (Scenario 2b)<br>(3a) Individual consumption data + range of use levels + occurence + 75% adherence);<br>(3b) Individual consumption data + Range of use levels + Occurence + 50% adherence
Ireland<br>Buffini *et al.*, 2017 [69]	Adults, 18–90 years (*n* = 1413) Participants of the National Adult Nutrition Survey (2011)	☒Ace-K<br>☒Aspartame<br>☒Cyclamate<br>☒Saccharin<br>☒Steviol<br>☒Sucralose<br>☐Thaumatin	4-day diary	☒MPL<br>☐Reported Use Level<br>☒Analytical Data (*n* = 377)<br>Collected from major Irish supermarkets	☒Yes<br>☐No<br>Irish National Food Ingredient Database V4 and food label survey	☐Yes<br>☒No	☐Deterministic<br>☒Simple Distribution<br>☐Probabilistic<br>3 scenarios:<br>(A) Individual consumption data + MPL<br>(B) Individual consumption data + MPL + occurence<br>(C) Individual consumption data + Actual concentration + occurence
Italy<br>Le Donne *et al.*, 2017 [66]	All ages, ≥3 years (*n* = 3,270) Participants of the INRAN-	☒Ace-K<br>☒Aspartame<br>☒Cyclamate<br>☒Saccharin	3-day diary	☒MPL<br>☐Reported Use Level<br>☒Analytical Data	☒Yes<br>☐No	☐Yes<br>☒No	☐Deterministic<br>☒Simple Distribution<br>☒Probabilistic

Country Reference	Population Group Examined (*n*)	Sweeteners Investigated	Food Consumption Data	Chemical Data			Assessment Model(s)
				Concentration Data [1]	Presence Data	Market Share Data	Details
	SCAI (2005–2006)	☒Steviol<br>☒Sucralose<br>☒Thaumatin		(*n* = 304 for stevia; *n* = 290 all other)<br><br>Steviol glycoside samples purchased in Rome; other sweeteners purchased in Italy and analyzed Janvier *et al.*, 2015	Food label survey conducted in Rome (2014)		3 scenarios:<br>(A) Individual consumption data + MPL<br>(B) Individual consumption data + MPL + occurence<br>(C) Individual consumption data + Range of concentrations + occurence
Ireland<br>O'Sullivan *et al.*, 2017 [67]	Young healthy children, 1.5–3 years (*n* = 2096), used as a surrogate for children with PKU<br>Participants of the UK NDNS (1992–1993; 2008–2010; 2011–2012)	☒Ace-K<br>☐Aspartame<br>☐Cyclamate<br>☐Saccharin<br>☐Steviol<br>☒Sucralose<br>☐Thaumatin	4-day food diary	☒MPL<br>☒Reported Use Level (FSMPs, provided by Nutricia)<br>☐Analytical Data	☒Yes<br>☐No<br><br>NHS Drug Tariff back issues, obtained from the British National Library Service (1992-2012)	☐Yes<br>☒No	☐Deterministic<br>☒Simple Distribution<br>☒Probabilistic<br><br>4 scenarios:<br>(A) MPL + 75% adherence<br>(B) MPL + 50% adherence<br>(C) Range of concentrations + occurence + 75% adherence<br>(D) Range of concentrations + occurence + 50% adherence

**Country Reference**	**Population Group Examined (*n*)**	**Sweeteners Investigated**	**Food Consumption Data**	**Chemical Data**			**Assessment Model(s)**
				**Concentration Data** [1]	**Presence Data**	**Market Share Data**	**Details**
EU Member States<br><br>Tennant and Bruyninckx, 2017 [68]	Infants, toddlers, other children, adolescents, adults, elderly, very elderly (*n* = NR)	☐Ace-K<br>☐Aspartame<br>☐Cyclamate<br>☐Saccharin<br>☒Steviol<br>☐Sucralose<br>☐Thaumatin	Range of dietary intake methodologies included in the EFSA Comprehensive European Food Consumption Database, Level 3 summary statistics	☒MPL<br>☐Reported Use Level<br>☐Analytical Data	☒Yes<br>☐No<br><br>Mintel Global New Products Database (2015)	☐Yes<br>☒No	☒Deterministic<br>☐Simple Distribution<br>☐Probabilistic<br><br>4 scenarios:<br>(A) Summary statistics + MPL<br>(B) Summary statistics + MPL + brand loyal<br>(C) Summary statistics + MPL + occurence<br>(D) Summary statistics + MPL + Occurence + brand loyal
EU Member States<br><br>EFSA, 2013 [70]	Toddlers, 12–35 months; Children, 3–9 years; Adolescents, 10–17 years; Adults, 18-64 years; Elderly, ≥65 years (*n* = NR)	☐Ace-K<br>☒Aspartame<br>☐Cyclamate<br>☐Saccharin<br>☐Steviol<br>☐Sucralose<br>☐Thaumatin	Range of dietary intake methodologies included in the EFSA Comprehensive European Food Consumption Database (2010) [79]	☒MPL<br>☒Reported Use Level<br>☒Analytical Data<br><br>Combination of industry use levels and analytical data	☐Yes<br>☒No	☐Yes<br>☒No	☒Deterministic<br>☐Simple Distribution<br>☐Probabilistic<br><br>2 scenarios:<br>(A) Summary statistics + MPL<br>(B) Summary statistics + actual concentration (reported/analytical)
EU Member States	Toddlers, 12–35 months; Children, 3–9	☐Ace-K<br>☐Aspartame<br>☐Cyclamate	Range of dietary intake methodologies	☒MPL + extension of use[6]	☐Yes<br>☒No	☐Yes<br>☒No	☒Deterministic<br>☐Simple Distribution<br>☐Probabilistic

Country Reference	Population Group Examined (*n*)	Sweeteners Investigated	Food Consumption Data	Chemical Data			Assessment Model(s)
				**Concentration Data** [1]	**Presence Data**	**Market Share Data**	**Details**
EFSA, 2015a [73]	years; Adolescents, 10–17 years; Adults, 18–64 years; Elderly, ≥65 years (*n* = NR)	☐Saccharin<br>☒Steviol<br>☐Sucralose<br>☐Thaumatin	included in the EFSA Comprehensive European Food Consumption Database (2010) [79]	☐Reported Use Level<br>☐Analytical Data			Summary statistics + MPL/extension of use
EU Member States<br>EFSA, 2015b [74]	Toddlers, 12–35 months; Children, 3–9 years; Adolescents, 10–17 years; Adults, 18–64 years; Elderly, ≥65 years (*n* = NR)	☐Ace-K<br>☐Aspartame<br>☐Cyclamate<br>☐Saccharin<br>☐Steviol<br>☐Sucralose<br>☒Thaumatin	FAIM tool (summary intakes)	☒MPL + extension of use [7]<br>☐Reported Use Level<br>☐Analytical Data	☐Yes<br>☒No	☐Yes<br>☒No	☒Deterministic<br>☐Simple Distribution<br>☐Probabilistic<br><br>Summary statistics + MPL/extension of use
Assessment from FSMPs in Young Children<br>EFSA, 2016a [75]	Young children, 1-3 years	☒Ace-K<br>☐Aspartame<br>☐Cyclamate<br>☐Saccharin<br>☐Steviol<br>☐Sucralose<br>☐Thaumatin	Fixed protein intake (absolute or bw)	Fixed value of 4.1, 5.9, or 9 mg ace-k/g protein	☐Yes<br>☒No	☐Yes<br>☒No	☒Deterministic<br>☐Simple Distribution<br>☐Probabilistic<br><br>6 scenarios:<br>(A) 9 mg ace-k/g protein, providing 20 g protein/day<br>(B) 9 mg ace-k/g protein, providing 10 g protein/day

Country Reference	Population Group Examined (*n*)	Sweeteners Investigated	Food Consumption Data	Chemical Data			Assessment Model(s)
				Concentration Data [1]	Presence Data	Market Share Data	Details
							(C) 5.9 mg ace-k/g protein, providing 3 g protein/kg bw/day<br>(D) 4.1 mg ace-k/g protein, providing 3 g protein/kg bw/day<br>(E) 5.9 mg ace-k/g protein, providing 20 g protein/ day<br>(F) 5.9 mg ace-k/g protein, providing 10 g proteinday
Assessment from FSMPs in Young Children<br>EFSA, 2016b [76]	Young children, 1–3 years	☐Ace-K<br>☐Aspartame<br>☐Cyclamate<br>☐Saccharin<br>☐Steviol<br>☒Sucralose<br>☐Thaumatin	Fixed protein intake (absolute or bw)	Fixed value of 4 mg sucralose/g protein	☐Yes<br>☒No	☐Yes<br>☒No	☒Deterministic<br>☐Simple Distribution<br>☐Probabilistic<br><br>3 scenarios, wherein there is 4 mg sucralose/g protein:<br>(A) Standard fixed protein intake of 14.5 g/day<br>(B) Maximum protein intake of 3 g/kg bw/day<br>(C) Protein intake ranging between 0.95 to 0.73 g /kg bw/day in children weighing between 10.2 to 14.7 kg

Ace-K = acesulfame-K; bw = body weight; CMPA = cow's milk protein allergy; DGCCRF = Directorate General for Fair Trading, Consumer Affairs and Fraud Control; DNFCS = Dutch National Food Consumption Survey; EFSA = European Food Safety Authority; EU = European Union; FAIM = Food Additive Intake Model; FFQ = food frequency questionnaire; FSAI = Food Safety Authority of Ireland; FSMP = foods for special medical purposes; HSE = Health Service Exceutive; INCA = Individual and National Study on Food Consumption; INRAN-SCAI = Italian National Food Consumption Survey; MPL = maximum permitted level; n = sample size; n/a = not applicable; NCFS = National Children's Food Survey; NDNS = National Diet and Nutrition Surveys; NHS = National Health Service; NPNS = National Pre-school Nutrition Survey; NR = not reported; NSIFCS = North/South Ireland Food Consumption Survey; NTFS = National Teens Food Survey; PKU = phenylketonuria; SFAE = French Association for Children's Food; T1D = type 1 diabetes; UK = United Kingdom. [1] MPL values for European studies obtained from Regulation 1333/2008 [77]. [2] Intake from food estimated for adults only. [3] Survey detail reported in Mischek [56] limited; survey details obtained from Elmadfa and Freisling (2004). [4] Assessment was also conducted using the FAIM template; however the authors noted that this database is not suitable for estimating exposures of young patients to artificial sweeteners from FSMPs because of the differences in dietary consumption patterns compared with healthy young children. [5] Data for scenarios 2a and 2b based on information from Table 2 in manuscript. [6] Extension of use of up to 29 mg/L steviol equivalents in tea, coffee, herbal infusion beverages, instant coffee and instant cappuccino products. [7] Extension of use of up to 5 mg/L thaumatin in flavored drinks. Extended condition of use as a flavor carrier. *Quantum satis* in all food flavorings.

Table S4. Methodologies Utilized for Intake Assessments Conducted for Low-/No-Calorie Sweeteners in Latin America.

Country Reference	Population Group Examined (n)	Sweeteners Investigated	Food Consumption Data	Chemical Data			Assessment Model(s)
				Concentration Data	Presence Data	Market Share Data	Details
Argentina Cagnasso *et al*., 2007 [81]	Cohort of children and adolescents attending public and private schools (middle and upper middle class) in Buenos Aires, 3–18 years (*n* = 190)	☒Ace-K ☒Aspartame ☒Cyclamate ☒Saccharin ☐Steviol ☐Sucralose ☐Thaumatin	FFQ of non-alcoholic beverages combined with portion size data (Technical Resolution MERCOSUR 47/03)	☐MPL ☒Reported use level ☐Analytical data<br><br>Concentration declared on beverage labels	☒Yes ☐No<br><br>Food label data	☐Yes ☒No	☐Deterministic ☒Simple Distribution ☐Probabilistic<br><br>Individual consumption data + use levels from food labels
Chile Durán Agüero *et al*., 2011 [82]	Cohort of children attending school in the Valparaíso region, 6–14 years (*n* = 281)	☒Ace-K ☒Aspartame ☒Cyclamate ☒Saccharin ☒Steviol ☒Sucralose ☐Thaumatin	FFQ of 122 food products containing artificial sweeteners collected from local supermarkets, , combined with household measures	☐MPL ☒Reported use level ☐Analytical data<br><br>Concentration declared on food labels	☒Yes ☐No<br><br>Food label data	☐Yes ☒No	☐Deterministic ☒Simple Distribution ☐Probabilistic<br><br>Individual consumption data + use levels from food labels

Country Reference	Population Group Examined (n)	Sweeteners Investigated	Food Consumption Data	Chemical Data			Assessment Model(s)
				Concentration Data	Presence Data	Market Share Data	Details
Chile<br>Hamilton *et al*., 2013 [85]	Cohort from the Metropolitan region involving Adults, 18–79 years (*n* = 477); children, 6–17 years (*n* = 516); adults with diabetes (Type1/2), 18–79 years (*n* = 155); children with diabetes (Type 1), 3–17 years (*n* = 63)	☒Ace-K<br>☒Aspartame<br>☒Cyclamate<br>☒Saccharin<br>☒Steviol<br>☒Sucralose<br>☐Thaumatin	FFQ of 207 food products containing artificial sweeteners, combined with food atlas of portion sizes collected from Chilean markets	☐MPL<br>☒Reported use level<br>☐Analytical data<br><br>Concentration declared on food labels	☒Yes<br>☐No<br><br>Food label data	☐Yes<br>☒No	☐Deterministic<br>☒Simple Distribution<br>☐Probabilistic<br><br>Individual consumption data + use levels from food labels
Chile<br>Durán Agüero *et al*., 2014 [83]	Cohort of school children from Viña del Mar and Santiago, 10–16 years (*n* = 571)	☒Ace-K<br>☒Aspartame<br>☒Cyclamate<br>☒Saccharin<br>☐Steviol<br>☒Sucralose<br>☐Thaumatin	FFQ (1 week) of 144 food products containing artificial sweeteners, combined with household measures	☐MPL<br>☒Reported use level<br>☐Analytical data<br><br>Concentration declared on food labels	☒Yes<br>☐No<br><br>Food label data	☐Yes<br>☒No	☐Deterministic<br>☒Simple Distribution<br>☐Probabilistic<br><br>Individual consumption data + use levels from food labels

**Country Reference**	**Population Group Examined (n)**	**Sweeteners Investigated**	**Food Consumption Data**	**Chemical Data**			**Assessment Model(s)**
				**Concentration Data**	**Presence Data**	**Market Share Data**	**Details**
Chile, Panama, Guatemala, and Peru<br><br>Durán Agüero *et al.*, 2015a [86]	Cohort of adults attending university from each country, 18-26 years (*n* = 1224)	☒Ace-K<br>☒Aspartame<br>☐Cyclamate<br>☐Saccharin<br>☐Steviol<br>☒Sucralose<br>☐Thaumatin	FFQ (1 week) of 122, 109, 29, and 124 food products containing artificial sweeteners, combined with household measures, collected from Chile, Panama, Guatemala, and Peru, respectively	☐MPL<br>☒Reported use level<br>☐Analytical data<br><br>Concentration declared on food labels	☒Yes<br>☐No<br><br>Food label data	☐Yes<br>☒No	☐Deterministic<br>☒Simple Distribution<br>☐Probabilistic<br><br>Individual consumption data + use levels from food labels
Chile, Panama, Guatemala, and Peru<br><br>Durán Agüero *et al.*, 2015b [87]	Cohort of adults attending university from each country, aged 18–26 years (*n* = 1229)	☒Ace-K<br>☒Aspartame<br>☐Cyclamate<br>☐Saccharin<br>☐Steviol<br>☒Sucralose<br>☐Thaumatin	FFQ (1 week) for carbonated beverage products containing artificial sweeteners , combined with food atlas of portion sizes	☐MPL<br>☒Reported use level<br>☐Analytical data<br><br>Concentration declared on food labels	☒Yes<br>☐No<br><br>Food label data	☐Yes<br>☒No	☐Deterministic<br>☒Simple Distribution<br>☐Probabilistic<br><br>Individual consumption data + use levels from food labels

Country Reference	Population Group Examined (n)	Sweeteners Investigated	Food Consumption Data	Chemical Data			Assessment Model(s)
				**Concentration Data**	**Presence Data**	**Market Share Data**	**Details**
Chile<br>Durán Agüero *et al*., 2015c [84]	Cohort of adults attending 4 different universities (first year students, mean age 20.3 to 20.8 years (*n* = 486)	☐Ace-K<br>☐Aspartame<br>☐Cyclamate<br>☐Saccharin<br>☒Steviol<br>☐Sucralose<br>☐Thaumatin	FFQ (1 week) of food and beverage products containing stevia	☐MPL<br>☒Reported use level<br>☐Analytical data<br><br>Concentration declared on food labels	☒Yes<br>☐No<br><br>Food label data	☐Yes<br>☒No	☐Deterministic<br>☒Simple Distribution<br>☐Probabilistic<br><br>Individual consumption data + use levels from food labels

Ace-K = acesulfame-K; FFQ= food frequency questionnaire; MPL = maximum permitted level; *n* = sample size; SD = standard deviation.

Table S5. Methodologies Utilized for Intake Assessments Conducted for Low-/No-Calorie Sweeteners by JECFA.

Country Reference	Population Group Examined (n)	Sweeteners Investigated	Food Consumption Data	Chemical Data			Assessment Model(s)
				Concentration Data	Presence Data	Market Share Data	Details
Global<br>JECFA, 2009 [17]	Global population; China; Japan; US	☐Ace-K<br>☐Aspartame<br>☐Cyclamate<br>☐Saccharin<br>☒Steviol<br>☐Sucralose<br>☐Thaumatin	GEMS/Food consumption database; *per capita* disappearance (China and Japan); *per capita* replacement (Japan, US); consumption data reported in children, and diabetic children/adults	Replacement of dietary sugars by stevia according to sweeteness ratio (1:200)	☐Yes<br>☒No	☐Yes<br>☒No	☒Deterministic<br>☐Simple Distribution<br>☐Probabilistic<br><br>In addition to published dietary exposure analyses
Global<br>JECFA, 2010 [92]	Global population	☐Ace-K<br>☐Aspartame<br>☒Cyclamate<br>☐Saccharin<br>☐Steviol<br>☐Sucralose<br>☐Thaumatin	Various dietary surveys conducted in Australia, Brazil, Germany, Italy, New Zealand, Spain, and the United Kingdom	☒MPL<br>☒Reported use level<br>☒Analytical data	☐Yes<br>☒No	☐Yes<br>☒No	Published dietary exposure analyses

Ace-K = acesulfame-K; GEMS = Global Environmental Monitoring System; JECFA = Joint FAO/WHO Expert Committee on Food Additives; NR = not reported; US = United States.
